# Multilayer Strategy for Photoelectrochemical Hydrogen Generation: New Electrode Architecture that Alleviates Multiple Bottlenecks

**DOI:** 10.1007/s40820-022-00822-8

**Published:** 2022-03-25

**Authors:** Selvaraj Seenivasan, Hee Moon, Do-Heyoung Kim

**Affiliations:** School of Chemical Engineering, Chonnam National University, 77 Yongbong-ro, Gwangju, 61186 Republic of Korea

**Keywords:** Atomic layer deposition, Bismuth sulfide, n-p junction, Photoelectrochemical, Nickel sulfide

## Abstract

Years of research have demonstrated that the use of multiple components is essential to the development of a commercial photoelectrode to address specific bottlenecks, such as low charge separation and injection efficiency, low carrier diffusion length and lifetime, and poor durability. A facile strategy for the synthesis of multilayered photoanodes from atomic-layer-deposited ultrathin films has enabled a new type of electrode architecture with a total multilayer thickness of 15–17 nm. We illustrate the advantages of this electrode architecture by using nanolayers to address different bottlenecks, thus producing a multilayer photoelectrode with improved interface kinetics and shorter electron transport path, as determined by interface analyses. The photocurrent density was twice that of the bare structure and reached a maximum of 33.3 ± 2.1 mA cm^−2^ at 1.23 V_RHE_. An integrated overall water-splitting cell consisting of an electrocatalytic NiS cathode and Bi_2_S_3_/NiS/NiFeO/TiO_2_ photoanode was used for precious-metal-free seawater splitting at a cell voltage of 1.23 V without degradation. The results and root analyses suggest that the distinctive advantages of the electrode architecture, which are superior to those of bulk bottom-up core–shell and hierarchical architectures, originate from the high density of active sites and nanometer-scale layer thickness, which enhance the suitability for interface-oriented energy conversion processes.

**Supplementary Information:**

The online version contains supplementary material available at 10.1007/s40820-022-00822-8.

## Highlights

- A multilayer architecture of layers with different functions alleviates bottlenecks in photoelectrochemical (PEC) hydrogen generation. Precise thickness control within a few nanometers defines each layer’s functionality.
- A Bi_2_S_3_/NiS/NiFeO/TiO_2_ photoanode had a photocurrent density of 33.3 mA cm^−2^ at 1.23 VRHE under AM 1.5 G illumination.
- Noble-metal-free seawater splitting was performed in an integrated PEC-electrocatalytic cell with an NiS electrocathode and Bi_2_S_3_/NiS/NiFeO/TiO_2_ photoanode.

**Supplementary Information:**

The online version contains supplementary material available at 10.1007/s40820-022-00822-8.

## Graphical abstract

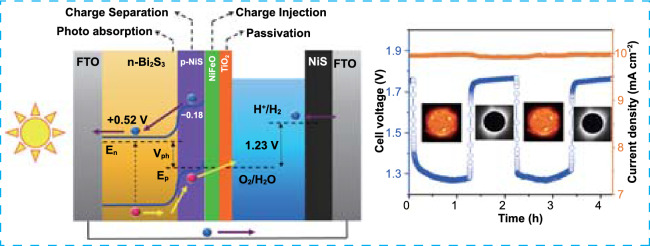

## Introduction

The generation of hydrogen, an ideal fuel, using photoelectrochemical (PEC) water splitting can establish a clean green energy source and alleviate the increasing energy crisis [1, 2]. The production of hydrogen by PEC water splitting is a subject of cutting-edge research that can ensure the stability of our ecosystem [3–5]. However, low solar-to-hydrogen (STH) efficiency and poor durability are the main barriers to the commercialization of the PEC electrodes reported to date [6, 7]. In addition, hydrogen generation should use nontoxic and Earth-abundant components to ensure affordability [8]. Highly active precious metal catalysts (such as IrO_2_ and Pt) are not appropriate candidates for replacing nonrenewable energy sources; the use of toxic elements requires more costly processing and disposal to maintain safety [9]. A light-absorbing semiconductor and surface catalyst are the main components of a PEC electrode. However, research has shown that several additional components, such as a heterojunction for effective charge separation and passivation layers for longer life, are essential to the development of commercial photoelectrodes [10–13]. Therefore, an appropriate choice of each component is critical for the full utilization of solar energy and for obtaining an efficient yield of light-induced charge carriers. Choosing narrow-band-gap semiconductors increases the likelihood of high charge carrier generation by extended light absorbance of solar spectrum. The formation of heterojunctions with opposite semiconductors is intensively studied due to the natural advantage of immense charge separation and long charge carrier lifetime. Furthermore, photocurrent generation is an interface-dominant electrochemical process that requires an extremely high density of surface-active sites, which accelerate the interface reactions to a sufficiently high rate. However, Effects of surface states (SS) are unavoidable, and surface recombination through SS greatly affects the net photocurrent at electrode‒electrolyte interfaces [14–17]. Surface recombination is responsible for more than 90% of the loss of light-induced charge carriers in various photoelectrodes. The integration of surface co-catalysts is the conventional method of alleviating the detrimental effects of SS and trapping charges at the interface before recombination. Therefore, the design of a co-catalyst within the thickness of the depletion region helps in an accurate analysis of the adaptive junctions that coexist with SS and the co-catalyst. Finally, passivation layers are essential for meeting industrial durability standards in large-scale applications.

Motivated by the above considerations, we designed a multilayer photoanode model in which each layer plays a different role, specifically, the primary light absorber, n-p junction, co-catalyst, and passivation layer, to obtain the maximum photocurrent output. The fabrication of the multilayer structure involves engineering difficulties such as the need to preserve the lower layers, selecting compatible synthesis methods, the buildup of solid–solid junction resistance, and, most importantly, avoiding the masking of primary light absorber. Therefore, we adopted atomic layer deposition (ALD) as the main tool to produce the multilayer structure because it can produce conformal thin films over high-aspect-ratio structures [18–20]. The dry, inert and high vacuum operating conditions of ALD make it possible to eliminate the engineering difficulties mentioned above in order to form multilayer electrodes as thin as possible. This model electrode differs from classical core–shell and hierarchical structures because it ensures multilayer formation within the band-bending region (5–10 nm) and, consequently, yields the fastest charge transfer kinetics. The use of atomically thin multilayers can decrease the migration distance of photogenerated charge carriers and increase the probability of carrier transfer at electrode‒electrolyte interfaces compared with that of the bulk counterpart [21].

Regarding material selection, in addition to well-studied inorganic metal oxides with band gaps above 2.0 eV, metal chalcogenides have recently shown great potential for hydrogen generation because of their large light absorption [22]. In particular, n-type bismuth sulfide (Bi_2_S_3_), which has a direct narrow band gap (1.30–1.35 eV), can harvest the full UV–visible (UV–vis) region of the solar spectrum [23, 24]. NiS (0.9–1.0 eV) was then selected as its p-type counterpart because its band edge positions are compatible with those of several well-developed semiconductors, including bismuth sulfide, and it is thus suitable for forming heterojunctions [25, 26]. The cascade alignment of band edge positions between bismuth sulfide and nickel sulfide semiconductors can produce photogenerated carriers moving in opposite directions, which result in high photocurrent and STH efficiency. The inherent problem of the low bandgap semiconductor is photocorrosion and could be alleviated by adopting a co-catalyst to transfer the accumulated charge carriers to the electrolyte before it oxidizes the primary light absorber. Transition metal oxides/hydroxides/oxyhydroxides exhibit the best catalytic activity in PEC water splitting [18, 27]. In particular, nickel- and iron-based oxyhydroxides are widely reported to exhibit successful charge tunneling at the interface. Our group recently reported the stoichiometric dependence of the NiFeOOH co-catalyst on hematite photoanodes in the charge tunneling process [18]. At the top of the electrode, a thin layer of titanium dioxide was used as a passivation layer to avoid direct contact of the electrolyte with the active components [28].

In this study, the Bi_2_S_3_/NiS/NiFeO/TiO_2_ multilayer photoanode was systematically analyzed to identify the role of each functional layer deposited on bismuth sulfide. In addition, the mechanism of inherent SS passivation and ladder-like hole transport from the valence band (VB) of bismuth sulfide to the electrolyte was elucidated through interface studies. Furthermore, seawater splitting by a precious-metal-free integrated PEC-electrocatalytic (EC) water-splitting cell with a Bi_2_S_3_/NiS/NiFeO/TiO_2_ photoanode and NiS electrocathode was demonstrated.

## Experimental

### Fabrication of Bi_2_S_3_/NiS/Ni_0.75_Fe_2.25_O_4_/TiO_2_ Photoanodes

#### ***Synthesis of Bi***_***2***_***S***_***3***_*** Photoanodes***

Bismuth sulfide nanostructures were grown by a previously reported two-step solvothermal process [29]. First, 0.05 M tin chloride and 0.1 M thioacetamide are dissolved in 40 mL of ethanol and maintained at 80 °C for 4 h with a cleaned fluorine-doped tin oxide (FTO) glass substrate in an air-tight reactor. The formed SnS_*x*_ films over FTO substrates were cleaned several times with ethanol and dried under N_2_ flow. Then, the SnS_*x*_ films were hydrothermally treated with a 0.04 M bismuth chloride acidic solution for 24 h at 100 °C to form Bi_2_S_3_. The dried samples were annealed under N_2_ atmosphere at 450 °C for 2 h.

#### ***Synthesis of Bi***_***2***_***S***_***3***_***/NiS Photoanodes***

NiO thin films were deposited using a homemade flow-type ALD reactor maintained at 175 °C and 800 mTorr. Argon (99.999%) was used as both the carrier (50 sccm) and purging (250 sccm) gas. Nickel cyclopentadienyl [Ni(Cp)_2_] (Sigma-Aldrich, USA) and ozone (5% in O_2_) were used in the ALD process as the nickel precursor and counter-reactant, respectively [30]. A single ALD cycle consisted of four steps: a 1.5 s Ni(Cp)_2_ pulse, a 30 s precursor purge, a 3 s ozone pulse, and a 60 s ozone purge. Before ALD was performed on the Bi_2_S_3_ nanostructures, the ALD conditions were optimized using single-crystalline p-type silica (100) flat substrates (LG Siltron, Korea). A dry sulfurization process was employed to drive the anion exchange reaction (AER) to convert NiO into NiS [31]. To obtain Bi_2_S_3_/NiS photoanodes, NiO thin films with various thicknesses were coated on the Bi_2_S_3_/FTO substrates; thermal vulcanization was then conducted by placing 2 g of S powder and the FTO/Bi_2_S_3_/NiO samples in the upstream and downstream parts of a tube furnace, respectively, and heating them to 350 °C under N_2_ atmosphere for 1 h.

#### ***Synthesis of Bi***_***2***_***S***_***3***_***/NiS/Ni***_***0.75***_***Fe***_***2.25***_***O***_***4***_*** Photoanodes***

Atomic-layered Ni_0.75_Fe_2.25_O_4_ (NiFeO) was deposited on the Bi_2_S_3_/NiS photoanodes following a procedure similar to that reported in our previous work [18]. The NiFeO thickness was controlled by varying the number of ALD supercycles.

#### ***Synthesis of Bi***_***2***_***S***_***3***_***/NiS/Ni***_***0.75***_***Fe***_***2.25***_***O***_***4***_***/TiO***_***2***_*** Photoanodes***

Titanium oxide was coated on the prepared Bi_2_S_3_/NiS/NiFeO samples via ALD using a homemade ALD system at 175 °C using tetrakis(dimethylamido)titanium (99.9%, UP Chemicals) and hydrogen peroxide (30 vol% in water, Sigma-Aldrich) as the reactants [32]. The thickness of the TiO_2_ films was controlled by varying the number of ALD cycles. The coated films were used directly for PEC analysis without any postheat treatment processes.

#### Synthesis of FTO/NiS Electrodes

NiO thin films (10 nm) were deposited on cleaned bare FTO substrates. A dry sulfurization process was then applied to drive the AER to convert NiO into NiS.

### Characterization

The crystallinity of each sample was analyzed using high-resolution X-ray diffraction (XRD) measurements (PANalytical) with a 3D-PIXcel detector and Cu Kα radiation at 60 kV and 55 mA. High-resolution X-ray photoelectron spectroscopy (XPS) was employed using Kα radiation and seven Channeltron detectors. The structural and morphological properties of the electrodes were studied using high-resolution scanning electron microscopy (HR-SEM; JEOL JSM-7500F) coupled with energy-dispersive X-ray spectroscopy at an acceleration voltage of 15 kV, and high-resolution transmission electron microscopy (HR-TEM, TECNAI G2 F20). UV–vis spectroscopy (VARIAN) was also used to observe the optical properties of the photoanodes using bare FTO as a reference. A time-resolved photoluminescence (TRPL) study was performed using an inverted-type scanning confocal microscope (MicroTime-200, Picoquant, Germany) with a 40 × objective. A single-mode pulsed diode laser (LDH-P–C-470, Picoquant, Germany) with a ~ 30 ps pulse width and 0.3 mW average power was used as an excitation source. A dichroic mirror (490 DCXR, AHF), two long-pass filters (HQ500lp, AHF; FEL600, Thorlabs), and a single-photon avalanche diode (PDM series, MPD) were used to collect the emission from the samples. Time-correlated single-photon counting was used to count the fluorescence photons. Fitting was done using a biexponential decay model.

### Photoelectrochemical Measurements

The PEC measurements were performed in the presence of 0.25 M Na_2_S (pH ~ 12.5) at ~ 25 °C. Ag/AgCl and Pt sheets were used as reference and counter electrodes, respectively. The measured potentials versus Ag/AgCl were converted to reversible hydrogen electrode (RHE) using the Nernst equation. A 300 W Xe lamp was used as the light source for an AM 1.5 G simulation, and the incident power density (100 mW cm^−2^) was calibrated using a standard silicon cell. The open-circuit voltage decay was measured using the same setup. Photoelectrochemical impedance spectroscopy (PEIS) measurements were made at an amplitude of 10 mV and frequencies ranging from 10^−2^ to 10^6^ Hz. Mott–Schottky analysis was conducted under dark conditions. The gas evolution during photolysis was analyzed using gas chromatography (074–594-P1E Micro GC Fusion, INFICON) in 0.25 M Na_2_SO_4_ (pH ~ 7.0) electrolyte.

## Results and Discussion

### Physical Characterization of Bi_2_S_3_/NiS/NiFeO

**Fig. 1:**
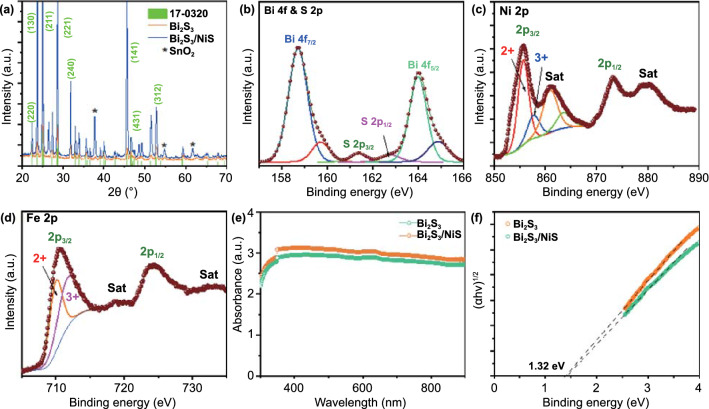
**a** XRD spectrum of Bi_2_S_3_ and Bi_2_S_3_/NiS photoanodes. High-resolution XPS spectra of Bi_2_S_3_/NiS/NiFeO photoanode: **b** Bi 4f and S 2p, **c** Ni 2p, and **d** Fe 2p. **e** UV–vis spectra of Bi_2_S_3_ and Bi_2_S_3_/NiS photoanodes measured with bare cleaned FTO as a reference to eliminate absorption by air and FTO. **f** Tauc plots of Bi_2_S_3_ and Bi_2_S_3_/NiS for direct allowed transitions

The XRD diffractograms of as-prepared Bi_2_S_3_ and Bi_2_S_3_/NiS are shown in Fig. 1a. All the diffraction peaks matched those of orthorhombic bismuthinite (JCPDS No. 017–0320), and no impurity phases were identified [23]. Furthermore, the sharp peaks that were observed in the diffraction pattern after the formation of NiS is due to annealing in the S environment indicated that the prepared Bi_2_S_3_ was very crystalline. No diffraction peaks corresponding to the NiS phase were observed due to the predominance of the large Bi_2_S_3_ core (~ 2 µm) over the NiS nanolayer (~ 5 nm). The surface composition of the Bi_2_S_3_/NiS/NiFeO photoanode was analyzed using XPS; peaks for the spin orbitals Bi 4f_7/2_ and Bi 4f_5/2_ appear at 158.7 and 164.0 eV, respectively, with a splitting energy of 5.3 eV (Fig. 1b) [33]. Two additional peaks corresponding to the S 2p_3/2_ and S 2p_1/2_ spin orbitals at 161.36 and 162.9 eV, respectively, overlap the Bi 4f peaks [34]. Figure 1c shows the high-resolution Ni 2p spectra, which indicate that most of the Ni atoms are in the + 2 oxidation state, as shown by the intense peak at 856.29 eV and its satellite peak at 861.50 eV. The peak at 859.13 eV indicates the coexistence of Ni^3+^ ions in the NiFeO co-catalyst layer. Figure 1d shows the high-resolution Fe 2p spectra, where the + 2 and + 3 oxidation states are represented by the intense peaks at 710.19 and 712.16 eV, respectively. The UV–vis absorption spectra of Bi_2_S_3_ and Bi_2_S_3_/NiS are shown in Fig. 1e, where both photoanodes exhibit strong light absorption in the full visible wavelength range because of the intrinsic narrow band gap of Bi_2_S_3_. There is a slight increase in light absorption, which was not significantly altered by increasing the thickness of the NiS nanoshell because the nanoshell thickness is negligible compared to the Bi_2_S_3_ core width. To further confirm the presence of the NiS phase, a detailed physical characterization of FTO/NiS is conducted and presented in Fig. S1. The absorbance is compared in Fig. S2a. A sharp increase in absorption appeared at 260 nm, and the absorption range covered the entire visible light region [35]. The Tauc plot of NiS indicates a band gap of approximately 1.01 eV, which is an intrinsic characteristic of NiS, as shown in Fig. S2b. The Tauc plots of Bi_2_S_3_ and Bi_2_S_3_/NiS for direct allowed transitions revealed a band gap of ~ 1.32 eV, as shown in Fig. 1f.

**Fig. 2:**
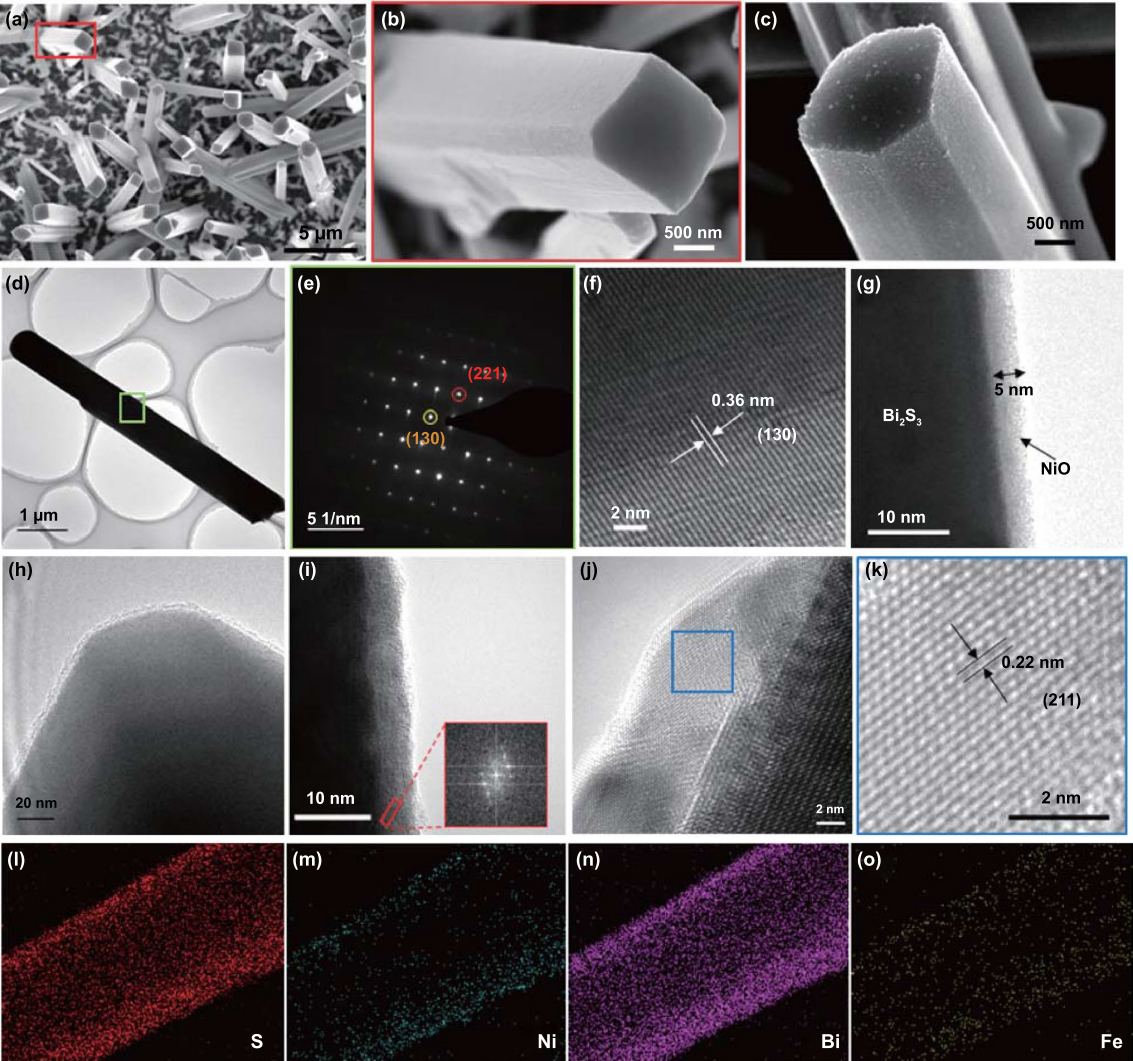
HR-SEM images of **a, b** Bi_2_S_3_ and **c** Bi_2_S_3_/NiS. **d** HR-TEM image of Bi_2_S_3_ and **e** SAED pattern of the area in the green square in **d**. **f** Diffraction plane analysis of Bi_2_S_3_. HR-TEM images of **g** Bi_2_S_3_/NiO and **h–k** Bi_2_S_3_/NiS (inset: FFT image). **l–o** Elemental mapping of Bi_2_S_3_/NiS/NiFeO photoanode

Figure 2a–c shows HR-SEM images of bismuth sulfide before and after the addition of the NiS layer (by the ALD and subsequent sulfurization of a 5 nm NiO layer). As shown in Figs. 2a and S3, the bismuth sulfide rods are vertically aligned and have a height and width of 10 and 1 μm, respectively. Magnified images of the bare and NiS-coated bismuth sulfide rods are shown in Fig. 2b–c, respectively; the drastic increase in surface roughness originates from the NiS nanoshell. The interface properties and the structural changes resulting from anion exchange were examined by HR-TEM analysis. An HR-TEM image of the bare bismuth sulfide rod is shown in Fig. 2d, and the ordered bright spots in the selected area electron diffraction (SAED) pattern indicate the high crystallinity and orientation of the bismuth sulfide rods, as shown in Fig. 2e. The higher-magnification image in Fig. 2f shows lattice fringes of 0.36 nm, which correspond to the (130) crystal plane of orthorhombic bismuth sulfide [24, 36]. The bismuth sulfide rod coated with the 5 nm ALD-deposited NiO film is shown in Fig. 2g. The uniformity of the NiO film demonstrates that ALD can be used to coat high-aspect-ratio nanostructures with excellent uniformity and conformality [37]. After the AER, a NiS shell with a thickness of 6–7 nm can be observed on the bismuth sulfide rods, as shown in Fig. 2h. The fast Fourier transform (FFT) image with ordered bright spots (inset in Fig. 2i) also confirms the crystalline nature of the shell material; thus, the NiS nanoshell can be expected to have an appropriate band gap and alignment with bismuth sulfide. The higher-magnification images in Fig. 2j–k reveal highly ordered lattice fringes with a *d* spacing of 0.22 nm, which corresponds to the (211) crystal plane of the NiS phase (1:1 stoichiometry) [38]. The elemental distributions of the Bi_2_S_3_/NiS/NiFeO photoanode (Fig. 2l–o) confirm the discrete distributions of elemental Ni and Fe over the bismuth sulfide rods, which clearly indicates continuous shell formation. Features corresponding to NiFeO are not visible because the deposited NiFeO films are ultrathin. The optimized NiFeO thickness, as indicated by the electrochemical performance, is 0.6–1.0 nm.

### Photoelectrochemical Performance

**Scheme 1:**
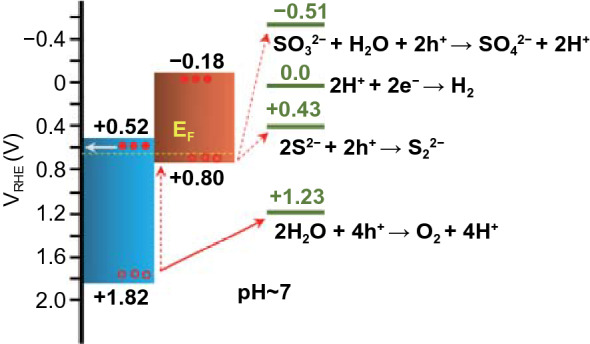
Band diagram of heterojunction, with band edges and chemical redox potentials of possible reactions with respect to RHE

**Fig. 3:**
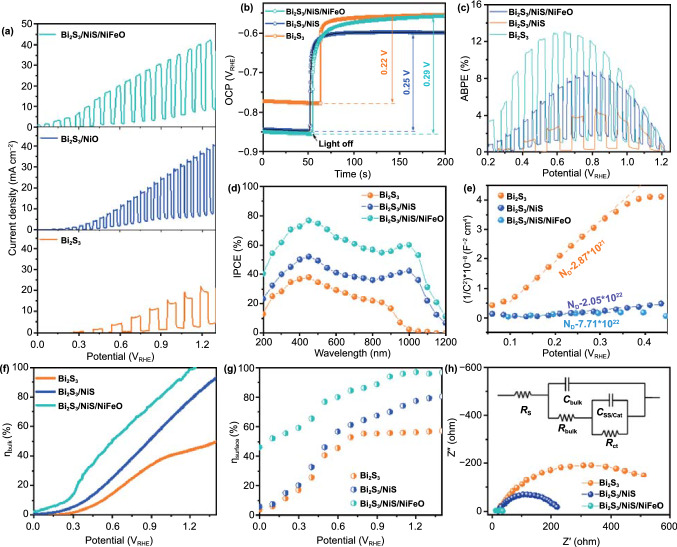
**a** *J*–*V* curve measured under AM 1.5 G illumination, **b** open-circuit decay curve, **c** ABPE analysis, **d** IPCE analysis, **e** Mott–Schottky plot using double-layer capacitance measured by dark EIS, **f** charge separation efficiency, and **g** charge injection efficiency. **h** Nyquist plot of photoanodes at 1.23 V_RHE_

The Bi_2_S_3_/NiS/NiFeO/TiO_2_ photoanode has three functional layers with a total thickness of 15–17 nm, which directly affect the electrochemical interfacial kinetics. The NiS nanoshell is the first functional nanoshell/nanolayer over the Bi_2_S_3_ primary light absorber. Both bismuth sulfide and nickel sulfide are narrow-band-gap semiconductors with opposite majority charge carriers, and they tend to form type-II cascade heterojunctions in equilibrium [39, 40]. The p-type behavior of NiS is confirmed by the *J*–*T* curve of the FTO/NiS photocathode at 0.0 V_RHE_ under chopped illumination (Fig. S4) [35]. Scheme 1 shows the band edge positions and the electrochemical potentials of possible interface reactions with respect to RHE at pH ~ 7. The Bi_2_S_3_/NiS photoanode generated a photocurrent density of 27.4 ± 1.5 mA cm^−2^ at 1.23 V_RHE_, which is more than twice that of the bare Bi_2_S_3_ photoanode, as shown in Fig. 3a. The nonzero dark current represents the formation of sulfite, as shown in Sch. 1. The sharp strike current of Bi_2_S_3_ and Bi_2_S_3_/NiS indicates a lack of surface activity and loss of charge carriers through surface recombination. When the second functional layer, the NiFeO co-catalyst, was added, the strike current disappeared, and the photocurrent density increased to 33.3 ± 2.1 mA cm^−2^ as a result of accelerated charge transfer from the VB of NiS to the electrolyte. The PEC performance of the Bi_2_S_3_/NiS/NiFeO photoanode is compared with the performance reported in recent studies in Table S1. Each nanoshell thickness is crucial to PEC water splitting because the individual electrocatalytic activity of NiS should not fade out the PEC activity of Bi_2_S_3_. The effect of nanoshell thickness was evaluated using *J*–*V* curves obtained under chopped illumination. As shown in Fig. S5, the NiS shell prepared from a 5-nm-thick NiO shell generates the maximum photocurrent density. The dark current increases with NiS shell thickness, indicating that the NiS electrocatalyst is becoming independent, which reduces the photocurrent density. The total current density (dark + light) across the NiS/electrolyte interface was compared with the charge transfer resistance (*R*_ct_) as shown in Fig. S5e. The *R*_*ct*_ increases slightly with increased shell thickness (10 nm) [41]. Therefore, the shell thickness was optimized as 5 nm, based on minimum *R*_*ct*_ and maximum photocurrent [31]. Furthermore, a control sample was synthesized by depositing a bulk NiS layer on Bi_2_S_3_ using the SILAR (Successive Ion Layer Reaction and Adsorption) method. The bulk NiS layer completely masks the PEC behavior as shown in Fig. S6. The effect of NiFeO thickness was evaluated using atomic thicknesses of 0.6, 1, 2, and 3 nm. The results (Fig. S7) indicate that a thickness of 1 nm is optimal for both catalytic activity and interfacial kinetics. The *R*_ct_ with respect to the different thicknesses of NiFeO follows a trend similar to that of the thickness of the NiS layer [42]. The open-circuit potential (OCP) decay curves in Fig. 3b show that *V*_ph_ significantly improved with the addition of the NiS and NiFeO functional layers. After the light source is turned off, the photoinduced electrons recombine with the holes or become trapped by SS [43]. The decay rate of the OCP to its dark equilibrium condition represents the life time of photoinduced electrons [44]. Bi_2_S_3_/NiS/NiFeO exhibits the slowest decay among the photoanodes, indicating reduced non-radiative recombination. The negative-shifted OCP of Bi_2_S_3_/NiS under dark condition is due to the detrimental potential drop across the Helmholtz layer [45]. The NiFeO co-catalyst incorporation successfully alleviated the potential drop and maintained the OCP at its original value. The photocurrent onset (*V*_on_) is a key indicator of surface catalyst efficiency; here, the 170 mV cathodic shift of Bi_2_S_3_/NiS after the addition of the NiS nanoshell originated from the intrinsic catalytic activity of Ni‒S active sites (Fig. S8a) [11]. The NiFeO co-catalyst provided a shift of ~ 250 mV in addition to that of Bi_2_S_3_/NiS; thus, the *V*_on_ value of Bi_2_S_3_/NiS/NiFeO is − 0.25 V_RHE_. *V*_on_ is generally influenced by two factors, the photovoltage (*V*_ph_) and kinetic overpotential (*η*), as follows: *E*_redox(dark)_ − *V*_on_ = *V*_ph_ − *η*, where *E*_redox(dark)_ is the electrochemical potential of the electrolyte under dark [46]. The obtained overall cathodic shift (~ 420 mV relative to *V*_on_ of bare Bi_2_S_3_) is lower than the increase in *V*_ph_ (~ 70 mV) measured under equilibrium conditions, as shown in Fig. 3b. Therefore, the obtained cathode shift can mainly be attributed to the decrease in kinetic overpotential as shown in Fig. S8b and Table S3. As shown in Fig. 3c, the highest applied bias photon-to-current efficiency (ABPE) is observed for Bi_2_S_3_/NiS/NiFeO (12.99% at 0.64 V_RHE_), followed by Bi_2_S_3_/NiS (6.56% at 0.80 V_RHE_) and Bi_2_S_3_ (4.48% at 0.82 V_RHE_). Figure 3d shows the incident photon to current conversion efficiency (IPCE) of bismuth sulfide photoanodes. The order of electrodes in terms of IPCE is as follows: Bi_2_S_3_/NiS/NiFeO (76.78%) > Bi_2_S_3_/NiS (52.16%) > Bi_2_S_3_ (36.16%) at 450 nm, and this corresponds to the order seen in the *J*–*V* curves. The similar shape of all IPCE spectra in the ultra-violet and visible region indicates the identical light absorption in corresponding region. The low IPCE in the ultra-violet and blue region represents the surface recombination effect. The declining IPCE response from the green to red region denotes the low diffusion length of charge carriers. The elevated IPCE response at the infrared region originates mainly from the photo absorbance of NiS in Bi_2_S_3_/NiS and Bi_2_S_3_/NiS/NiFeO photoanodes.

Mott–Schottky analysis was conducted in dark conditions, and the carrier density was found to be 2.87 × 10^21^ and 20.58 × 10^21^ cm^−3^ for Bi_2_S_3_ and Bi_2_S_3_/NiS, respectively, as shown in Fig. 3e. This drastic enhancement provides strong evidence for increased charge separation at the interface due to n-p junction formation. The addition of the NiFeO co-catalyst also increases the carrier concentration by ~ 3.5 times because the rapid tunneling of photogenerated charges through NiFeO reduces the probability of charge recombination. The yield of photogenerated charges at the interface (*η*_bulk_) for different Bi_2_S_3_ and Bi_2_S_3_/NiS samples was calculated using the procedures described in previous reports (Fig. S9). The calculated values of *η*_bulk_ for all the samples are plotted in Fig. 3f. The *η*_bulk_ value of Bi_2_S_3_/NiS is greater than that of Bi_2_S_3_ across the potential window. In particular, the more significant difference in *η*_bulk_ at higher potentials (> 0.9 V) indicates that NiS contributes mainly to charge separation, not tunneling. Although NiS is a well-known oxygen evolution reaction catalyst, the low *η*_bulk_ at lower potentials and large strike currents indicate the presence of SS on NiS, which is also reflected in the low *η*_surface_, as shown in Fig. 3g. The NiFeO co-catalyst increases *η*_bulk_, which reaches 100% at 1.2 V_RHE,_ by reducing charge recombination through rapid charge removal from the VB of NiS. The larger increase in *η*_surface_ at lower potentials (< 0.6 V_RHE_) when the NiFeO co-catalyst is present demonstrates accelerated charge tunneling at the NiFeO‒electrolyte interface. Nyquist plots of the Bi_2_S_3_, Bi_2_S_3_/NiS, and Bi_2_S_3_/NiS/NiFeO photoanodes under illumination were obtained at 1.23 V_RHE_, as shown in Fig. 3h. The drastic decrease in *R*_ct_ indicates accelerated charge tunneling at the NiS‒electrolyte and NiFeO‒electrolyte interfaces for the Bi_2_S_3_/NiS and Bi_2_S_3_/NiS/NiFeO photoanodes, respectively.

### Charge Transfer and Recombination Kinetics

**Fig. 4:**
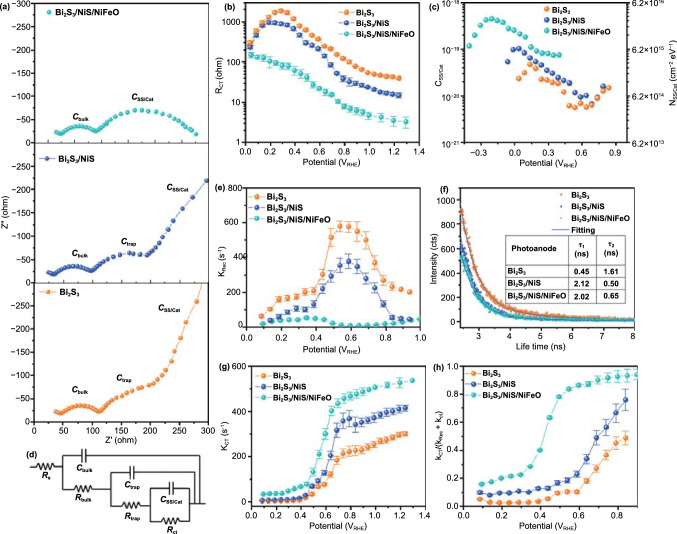
**a** Nyquist plot of bismuth sulfide photoanodes at 0.30 V_RHE_, **b** interface charge transfer resistance (*R*_ct_), and **c** distribution of *C*_SS/cat_ and *N*_SS_ calculated from PEIS measurements at various potentials under illumination in 0.25 M Na_2_S electrolyte, **d** Electronic circuit used to fit the PEIS data, **e** charge recombination rate constant (*k*_rec_), **f** TRPL transients, where solid blue line represents a bi-exponential fitting (table: fitted values), **g** charge transfer rate constant (*k*_ct_), and **h** overall quantum efficiency of photoanodes

The charge carrier kinetics were studied through the PEIS measurements with linear bias interval. The Nyquist plot of Bi_2_S_3_/NiS/NiFeO at 0.30 V_RHE_ has only two semicircles, which correspond to the capacitance of the bulk space-charge region/Helmholtz layer (*C*_bulk_) and charge tunneling (*C*_SS/cat_). Both the Bi_2_S_3_ and Bi_2_S_3_/NiS photoanodes show three semicircles, including *C*_trap_ in the medium-frequency range, which can be attributed to reaction intermediates or trap surface states (Fig. 4a). The disappearance of *C*_trap_ in Bi_2_S_3_/NiS/NiFeO indicates the successful passivation of surface traps by the NiFeO co-catalyst [47]. The *R*_ct_ values were calculated from the Nyquist plot by fitting the data using the equivalent circuit (in Fig. 4d), as shown in Fig. 4b. Bi_2_S_3_ shows the highest *R*_ct_ throughout the potential window. This result highlights the high density of surface traps on the Bi_2_S_3_ surface, which causes high charge recombination and thus low *η*_surface_; the photogenerated charges can overcome the trapping barrier only at high applied potentials. The addition of NiFeO decreases *R*_ct_ by two orders of magnitude, demonstrating accelerated charge tunneling through NiFeO. *C*_SS/cat_ decreases gradually with increasing bias for all the photoanodes owing to successful band bending and the release of accumulated charges to the electrolyte (Fig. 4c). The SS distribution was calculated from *C*_SS/cat_ as a function of applied potential (*E*) as follows [48]:

1 $$N_{{{\text{ss}}}} \left( E \right) = \frac{{C_{{{\text{SS}}/{\text{cat}}}} \left( E \right)}}{q}$$

where *q* is the electron charge (1.602 × 10^−19^ C).

The *N*_SS_ distribution is dominant for the Bi_2_S_3_/NiS/NiFeO photoanode because of the increased carrier density at the NiFeO/electrolyte interface, which is identical to the carrier density measured by Mott‒Schottky analysis. The shift in the maximum *N*_SS_ coincides with *V*_on_ for all the photoanodes. This result shows that charge tunneling in the Bi_2_S_3_/NiS and Bi_2_S_3_/NiS/NiFeO photoanodes occurs primarily through the SS of NiS and the NiFeO co-catalyst (Fig. S10). Although the SS are considered to be hot spots for surface recombination, the SS and catalysts are charged together and reach quasi-equilibrium, resulting in the passivation of the SS. [49] The charge transfer step can be quantitatively described by the interfacial charge transfer rate constant (*k*_ct_) derived from *R*_ct_. Similarly, the surface recombination can be quantified by the recombination rate constant (*k*_rec_), which is derived from *R*_trap_. Both rate constants were calculated using the PEIS data as follows [50, 51]:

2 $$k_{{{\text{ct}}}} = \frac{1}{{R_{{{\text{ct}}}} C_{{{\text{SS}}/{\text{cat}}}} }}$$

3 $$k_{{{\text{rec}}}} = \frac{1}{{R_{{{\text{trap}}}} C_{{{\text{SS}}/{\text{cat}}}} }}$$

In agreement with the *J*–*V* curve, the *k*_rec_ value of the Bi_2_S_3_ anode is higher, which results in low net photocurrent flow across the interface. The nonlinear behavior of the *k*_rec_ values of both the Bi_2_S_3_ and Bi_2_S_3_/NiS anodes indicates that band bending is modulated by the time dependened concentration of trapped holes at the SS [52]. The high concentration *N*_ss_ results in charge storage and modifies the potential drop across the Helmholtz layer. However, after the NiS nanoshell layer was added, *k*_rec_ was significantly reduced throughout the potential window, especially at higher potential (≥ 0.8 V_RHE_), as shown in Fig. 4e. These results are consistent with the *J*–*V* curves and *η*_bulk_ plots. Overall, the results suggest that the Bi_2_S_3_/NiS anode is severely affected by the SS at the interface, which decrease the benefits of n-p junction formation at lower potentials. Although the continuous forward polarization charges all the SS, further band bending (the absence of Fermi-level pinning) allows for the release of charges into the electrolyte. By contrast, the Bi_2_S_3_/NiS/NiFeO anode shows a monotonic decrease in *k*_rec_ with potential, indicating less dependence on the applied potential. This result is consistent with the shift in *Nss* maximum to lower potentials could charge the SS at lower potentials or the estimated high *Nss* values originally originates from surface-bounded reaction intermediates. The decrease in *k*_rec_ from Bi_2_S_3_ to Bi_2_S_3_/NiS/NiFeO ultimately increases the carrier lifetime. Quantitative measurement of the charge carrier lifetime can potentially be employed to evaluate the degree of charge separation. TRPL spectroscopy was used to determine the lifetime of excited carriers in bismuth sulfide before and after the formation of the n-p junction. The charge dynamics curves were fitted with a bi-exponential decay function (*i* = 2),

4 $$I\left( t \right) = \mathop \sum \limits_{i} A_{i } e^{{\left( {{\raise0.7ex\hbox{${ - t}$} \!\mathord{\left/ {\vphantom {{ - t} {\tau_{i} }}}\right.\kern-\nulldelimiterspace} \!\lower0.7ex\hbox{${\tau_{i} }$}}} \right)}}$$

where *I*(*t*) is the TRPL intensity, *A* is the amplitude as a normalized percentage, and *τ* is the lifetime [53]. The fitted results are shown in Fig. 4f; *τ*_*1*_ represents the non-radiative decay of charge carriers generated in the Bi_2_S_3_ core through the SS. Upon incorporation of NiS (n-p junction), *τ*_*1*_ increased drastically due to the alleviation of all SS by the electronic equilibrium attained at the Bi_2_S_3_/NiS interface [43, 54, 55]. Instead of being trapped by SS and recombined, the charge carriers are separated and accumulated at the valance band of NiS and conduction band of Bi_2_S_3_. The reduction in *τ*_*2*_ represents the successful extraction of charge carriers from NiS due to the creation of additional charge relaxation pathways [56, 57]. A carrier lifetime on the nanosecond scale is generally attributed to a short hole diffusion length (the maximum distance traveled by a carrier without recombination), that is, fast recombination. In real-time operation, the radiative and non-radiative lifetimes of charge carriers will be different or long as the photoelectrode will be connected in a complete electrical circuit with back contact and electrolyte to naturally consume the charge carriers. The carrier lifetime could also vary according to the changes in band edge positions upon polarization [58]. In non-contact TRPL analysis, the charge carriers quickly reach their original positions relative to real-time operating conditions when photon energy stops. However, this analysis gives reliable information about photoelectrodes that belong to the same sub-groups. The PL spectra of Bi_2_S_3_ shown in Fig. S11 have two main recombination pathways through the intermediate trap stats at 516.47 and 699.22 nm. The incorporation of NiS and NiFeO drastically reduces the fluorescence intensity by relieving recombination, as shown by the TRPL analysis. By contrast, *k*_ct_ is higher throughout the potential range compared to that of Bi_2_S_3_/NiS because NiFeO has a lower stoichiometric Ni^2+^ content and better catalytic properties than NiS (Fig. 4g). The shape of the *k*_ct_ curve is consistent with that of the *J*–*V* curve; briefly, the increase in photocurrent in Bi_2_S_3_/NiS/NiFeO is larger at lower potentials and becomes almost the same as that of Bi_2_S_3_/NiS at higher potentials. This phenomenon, a light-intensity-dependent photocurrent, is caused by extreme band bending. The depletion region extends beyond the n-p junction and could extract holes from the VB of bismuth sulfide directly. Similar phenomena also appear in the *J*–*V* curve of the Bi_2_S_3_/NiFeO photoanode (Fig. S12). Furthermore, the carrier lifetime increased from 1.10 to 1.54 ns because the presence of NiFeO sharply decreases *k*_rec_. From the obtained PEIS parameters, the overall quantum efficiency of all the photoanodes was calculated as follows:

5 $$\varphi = \frac{{k_{{{\text{ct}}}} }}{{k_{{{\text{rec}} + }} k_{{{\text{ct}}}} }}$$

Figure 4h plots the quantum efficiency versus potential; the curve is in excellent agreement with the *J*–*V* curve displayed in Fig. 3a. Specifically, *k*_ct_ is the dominant factor determining the shape of the *J*–*V* curve. As stated above, the enhanced PEC performance results from the formation of a multilayer structure with the necessary components (n-p junction, co-catalyst, and passivation layer) [59, 60].

**Fig. 5:**
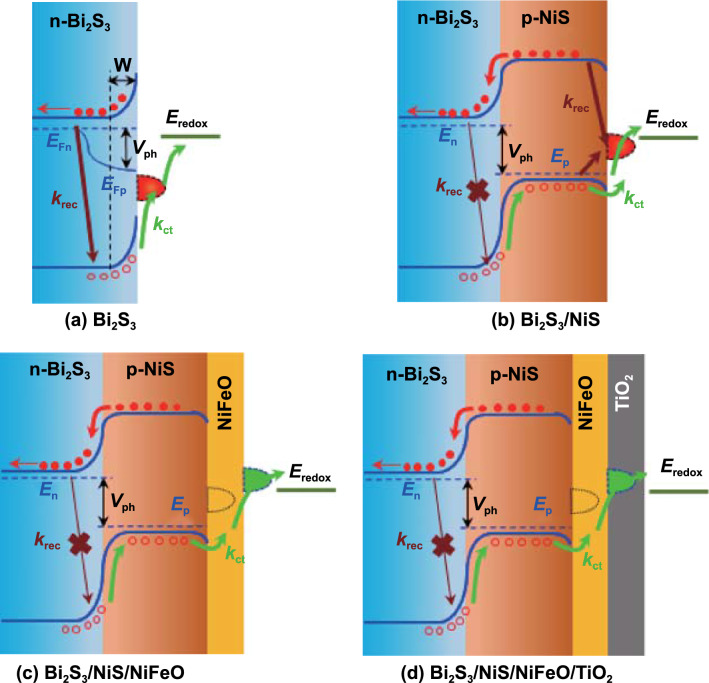
Band alignment of **a** bare Bi_2_S_3_, **b** Bi_2_S_3_/NiS, **c** Bi_2_S_3_/NiS/NiFeO, and **d** Bi_2_S_3_/NiS/NiFeO/TiO_2_ under forward bias. At equilibrium, the Fermi levels are aligned by electron sharing, which forms the internal field. Electrons and holes are spatially separated, and the maximum *V*_ph_ is limited by the positions of the Fermi levels of bismuth sulfide (*E*_n_) and nickel sulfide (*E*_p_). *E*_Fn_: electron Fermi level, *E*_Fp_: quasi-hole Fermi level

From the above results, the following working mechanism is proposed for the multilayer Bi_2_S_3_/NiS/NiFeO/TiO_2_ photoanodes for PEC hydrogen generation. The band alignments of the photoanodes under a forward bias in the absence of Fermi-level pinning are shown in Fig. 5a-b [61]. The Fermi level (*E*_F_) of bare Bi_2_S_3_ is in equilibrium with the redox potential; both the VB and conduction band (CB) undergo upward band bending within the space-charge region, which is labeled W, and the magnitude of band bending is controlled by the forward bias and the corresponding alignment of *E*_F_. Under illumination, the photoanode produces an inherent photovoltage (*V*_ph_) of 222 mV, which drives the holes into the electrolyte and results in upward shifts of the CB and VB edges, as shown in Fig. 5a [53, 62]. This potential is sufficiently weak to allow effortless charge recombination at a higher rate (*k*_rec_); as a result, only a fraction of the generated holes tunnel into the electrolyte. When the n-p junction is formed between Bi_2_S_3_ and the NiS nanolayer, an interior electric field is established because of electron sharing at the junction (Fermi-level equilibrium), as shown in Fig. 5b. The electric field causes spatial charge separation, which causes holes to collect in the VB of NiS and electrons to collect in the CB of Bi_2_S_3_. However, the existing SS comprise the actual port for charge tunneling, which consumes the photogenerated holes for charging as well as the forward bias. This significantly degrades the utilization of the n-p junction at lower bias, as indicated by the *J*–*V* curves and *η*_bulk_ plots. An ultrathin NiFeO co-catalyst layer effectively passivates the intrinsic SS in NiS and accelerates charge tunneling through the NiFeO itself, as shown in Fig. 5c. It drastically reduces the probability of recombination, which increases the carrier lifetime and ultimately the charge injection. Briefly, the multilayer configuration reduces the minimum bias required for charge tunneling by shifting the active SS to lower potential. Finally, the TiO_2_ passivation layer on the outside of the electrode prevents the physical degradation of the active layers in the multilayer photoanode. The effects of the TiO_2_ passivation layer on the durability and interfacial kinetics are discussed in the next section. This unique thin-film multilayer strategy for efficient electrochemical interface kinetics is potentially useful for many types of electrochemical energy conversion and storage applications.

### Integration of Electrocatalytic and Photoelectrochemical Systems for Noble-metal-free Water Splitting

**Fig. 6:**
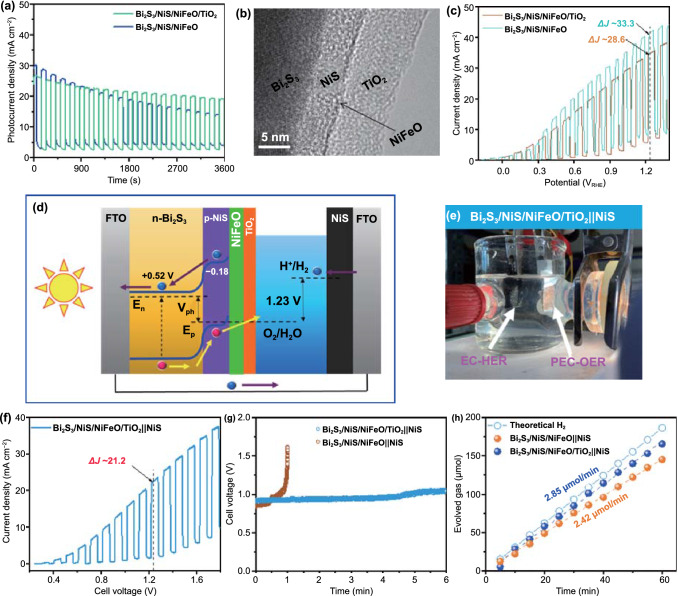
**a** *J*–*T* curves of Bi_2_S_3_/NiS/NiFeO and Bi_2_S_3_/NiS/NiFeO/TiO_2_ photoanodes under chopped illumination, **b** HR-TEM image of Bi_2_S_3_/NiS/NiFeO/TiO_2_ photoanode, **c** *J*–*V* curves before and after addition of TiO_2_ buffer layer, **d, e** two-electrode PEC-EC cell configuration with Bi_2_S_3_/NiS/NiFeO/TiO_2_ and NiS as photoanode and electrocathode, respectively, **f** *J*–*V* curve and **g** *J*–*T* curve of PEC-EC cell, **h** comparison of experimental H_2_ evolution at a current density of 10 mA cm^−2^

We verified the durability of the Bi_2_S_3_/NiS/NiFeO photoanode using chronoamperometry (CA) at a potential of 1.0 V_RHE_ under chopped illumination. The *J*–*T* curve in Fig. 6a shows that the Bi_2_S_3_/NiS/NiFeO photoanode retained only 58.35% of its initial photocurrent after 1 h of operation due to the degradation of NiS and Bi_2_S_3_ during prolonged high-current–density operation. A thin layer of TiO_2_ (~ 10 nm) was deposited over the Bi_2_S_3_/NiS/NiFeO photoanode as third functional layer to avoid direct contact with the electrolyte and maintain the structural integrity during high-current–density operation. The HR-TEM image in Figs. 6b and S13 shows clear boundaries between the Bi_2_S_3_ core, NiS nanoshell, NiFeO co-catalyst, and TiO_2_ buffer layer. In particular, a dark line between NiS and TiO_2_ with an approximate thickness of 1 nm represents conformal deposition of the NiFeO co-catalyst. The *J*–*V* curve of the Bi_2_S_3_/NiS/NiFeO photoanode before and after the addition of the TiO_2_ buffer layer is shown in Fig. 6c. The small decrease in photocurrent represents increased *R*_ct_ at the electrode‒electrolyte interface. The Nyquist plot in Fig. S14 shows that the higher *R*_ct_ values originate from TiO_2_ passivation layer. However, the Bi_2_S_3_/NiS/NiFeO/TiO_2_ photoanode showed significantly improved durability and retained 89.45% of its initial photocurrent after 1 h of operation.

The Pt electrocathode was replaced with the NiS electrocathode to make an integrated PEC-EC water-splitting cell for noble-metal-free water splitting, as shown in Fig. 6d-e. The individual electrocatalytic activity of NiS in the hydrogen evolution half-cell is shown in Fig. S15. As shown in Fig. 6f, the Bi_2_S_3_/NiS/NiFeO/TiO_2_||NiS PEC-EC cell achieved a current density of 21.2 mA cm^−2^ with zero bias (cell voltage, 1.23 V). The *J*–*V* curves of the Bi_2_S_3_/NiS/NiFeO/TiO_2_ photoanode with Pt and NiS as the electrocathode are shown in Fig. S16. Both electrodes provided identical *J*–*V* curves without an overpotential difference to facilitate water oxidation. Chronopotentiometry studies of the Bi_2_S_3_/NiS/NiFeO/TiO_2_||NiS cell were conducted in an air-tight H cell where both electrodes are separated by a Nafion membrane (Fig. S17a). The *J-T* curve shows a slight increase in applied potential to maintain a photocurrent density of 10 mA cm^−2^ during 6 h of operation, Fig. 6g. The *J*–*T* curve of the Bi_2_S_3_/NiS/NiFeO||NiS cell largely deviates from a straight *J*–*T* profile owing to the detachment of the Bi_2_S_3_/NiS/NiFeO photoanode from the FTO substrate as shown in Fig. S17b. To accurately measure the amount of H_2_ generated, the experiments were performed by connecting the air-tight H cell directly to a gas chromatography instrument with a N_2_ (20 sccm) purge. The average yields of H_2_ are shown in Fig. 6h. The H_2_ evolution rate was 2.85 and 2.42 µmol min^−1^ for the Bi_2_S_3_/NiS/NiFeO/TiO_2_||NiS and Bi_2_S_3_/NiS/NiFeO||NiS cells, respectively.

**Fig. 7:**
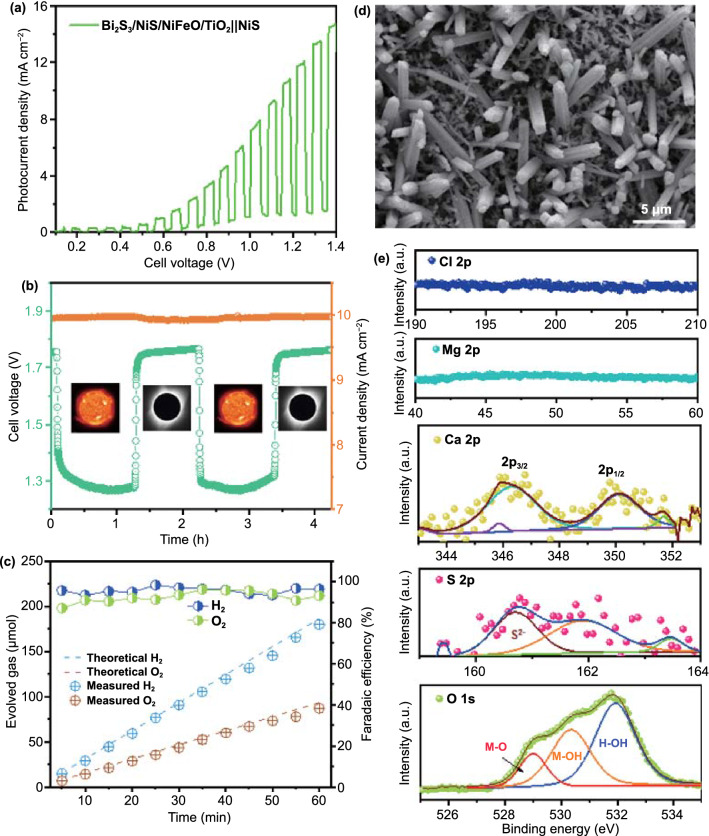
**a** *J–V* curve of integrated PEC-EC electrolyzer in seawater electrolyte under chopped illumination, **b** cell voltage measured under photo-electrolysis condition, **c** comparison of experimental H_2_ and O_2_ evolution at a current density of 10 mA cm^−2^ and calculated faradaic efficiency, **d** HR-SEM image and, **e** XPS spectra of Bi_2_S_3_/NiS/NiFeO/TiO_2_ photoanode after 4 h photo-electrolysis

Finally, seawater splitting experiments were conducted using the same two-electrode H-cell setup with 0.5 M Na_2_SO_4_ as a supporting electrolyte (pH ~ 7.5). The seawater was used after simple filtration to remove debris and fine sand particles without any chemical treatment (Gamami Beach, Jeollanam-do, South Korea). The *J*–*V* curve of the seawater photoelectrolyzer is shown in Fig. 7a. A photocurrent of 10.44 mA cm^−2^ was achieved at 0 V bias (cell voltage, 1.23 V) for seawater splitting at neutral pH. The photo-electrolysis experiment was conducted continuously for 4 h at a current density of 10 mA cm^−2^. The measured cell voltage under dark and illumination conditions is shown in Fig. 7b. The *V*_ph_ generated to maintain the operating current density is 463 mV, which is almost double the *V*_ph_ measured in the three electrode configuration. The cell voltage maintained without any increase during 4 h of photo-electrolysis revealed that the TiO_2_ passivation layer served the cause well. The H_2_ evolution rate (2.71 µmol min^−1^) is almost twice that of O_2_ (1.22 µmol min^−1^) in seawater electrolyte, as shown in Fig. 7c. The faradaic efficiencies exceeded 96.8% and 93.4% for H_2_ and O_2_, respectively, indicating that the selectivity of the photoanode is good even with multiple ions present in the seawater electrolyte. The HR-SEM image of Bi_2_S_3_/NiS/NiFeO/TiO_2_ photoanode after 4 h of photo-electrolysis does not show any structural degradation as shown in Fig. 7d. The direct use of seawater (pH ~ 8.1) leads to the formation of Mg(OH)_2_ and Ca(OH)_2_ by reactions with OH^−^ ions generated during electrolysis. Furthermore, the HCO_3_^−^ bicarbonate ions may react with OH^−^ ions and then, with Ca^2+^ ions to form insoluble CaCO_3_ particles [63]. These particles could be adsorbed on the electrodes and hinder the surface reactions. However, no trace of Mg and Cl was found as shown in Fig. 7e. It shows that the TiO_2_ surface is non-porous to allow the binding of excess inorganic chloride (NaCl) in the seawater electrolyte thanks to the ALD process. The 2p_3/2_ and 2p_1/2_ spin orbitals at 346.30 and 350.12 eV in the high-resolution Ca 2p profile represent the adsorption of Ca derivatives on the OER electrode. The atomic percentage of Ca was not significant, and no increase in cell voltage was observed; therefore, binding of Ca derivatives does not affect the electrode activity. The presence of S^2−^ (160.70 eV) and M–O (528.99 eV) peaks in the corresponding S 2p and O 1 s spectra confirms that the surface reconstruction of NiS and NiFeO to corresponding oxyhydroxides is largely prohibited by the TiO_2_ passivation layer [64]. The band alignment between Bi_2_S_3_ and NiS is well preserved. The M-OH peak (530.35 eV) indicates surface attached species from atmosphere or partial conversion of NiFeO during long-term photo-electrolysis.

## Conclusion

A novel multilayer strategy for constructing a robust photoelectrode structure for PEC water splitting was reported. Each functional layer was carefully crafted by nanometer-scale ALD to precisely control the functionality and maintain the shortest electron transport path. The final multilayer Bi_2_S_3_/NiS/NiFeO/TiO_2_ photoanode exhibited a photocurrent density of 33.3 ± 2.1 mA cm^−2^ at 1.23 V_RHE_ under AM 1.5 G illumination. Electrochemical interfacial analyses showed that each functional layer in the multilayer assembly played distinct roles in alleviating specific bottlenecks. Finally, noble-metal-free seawater splitting was demonstrated in an integrated PEC-EC cell using NiS as the electrocathode and a Bi_2_S_3_/NiS/NiFeO/TiO_2_ photoanode. The multilayer strategy will expand the options for improving interface-dominant electrochemical processes using revolutionary nanoshell architectures, which will contribute to the rational design and development of electrodes for energy conversion and storage.

## Supplementary Information

Below is the link to the electronic supplementary material.

Supplementary file1 (PDF 1600 kb)
