# Supplementary material for: Multilayer Strategy for Photoelectrochemical Hydrogen Generation: New Electrode Architecture that Alleviates Multiple Bottlenecks
Source: Nanomicro Lett. 2022 Mar 25;14:78. doi: 10.1007/s40820-022-00822-8 (PMC8956779; doi:10.1007/s40820-022-00822-8)
Supplement: Supplementary file 1 — Supplementary file1 (PDF 1600 kb) [file 40820_2022_822_MOESM1_ESM.pdf]

Supporting Information for

Multilayer Strategy for Photoelectrochemical Hydrogen Generation: New Electrode Architecture that Alleviates Multiple Bottlenecks

Selvaraj Seenivasan[1], Hee Moon[1], and Do-Heyoung Kim[1,]*

[1]School of Chemical Engineering, Chonnam National University, 77 Yongbong-ro, Gwangju 61186, Republic of Korea

*Corresponding author. E-mail: kdhh@chonnam.ac.kr (Do-Heyoung Kim)

Supplementary Figures and Tables

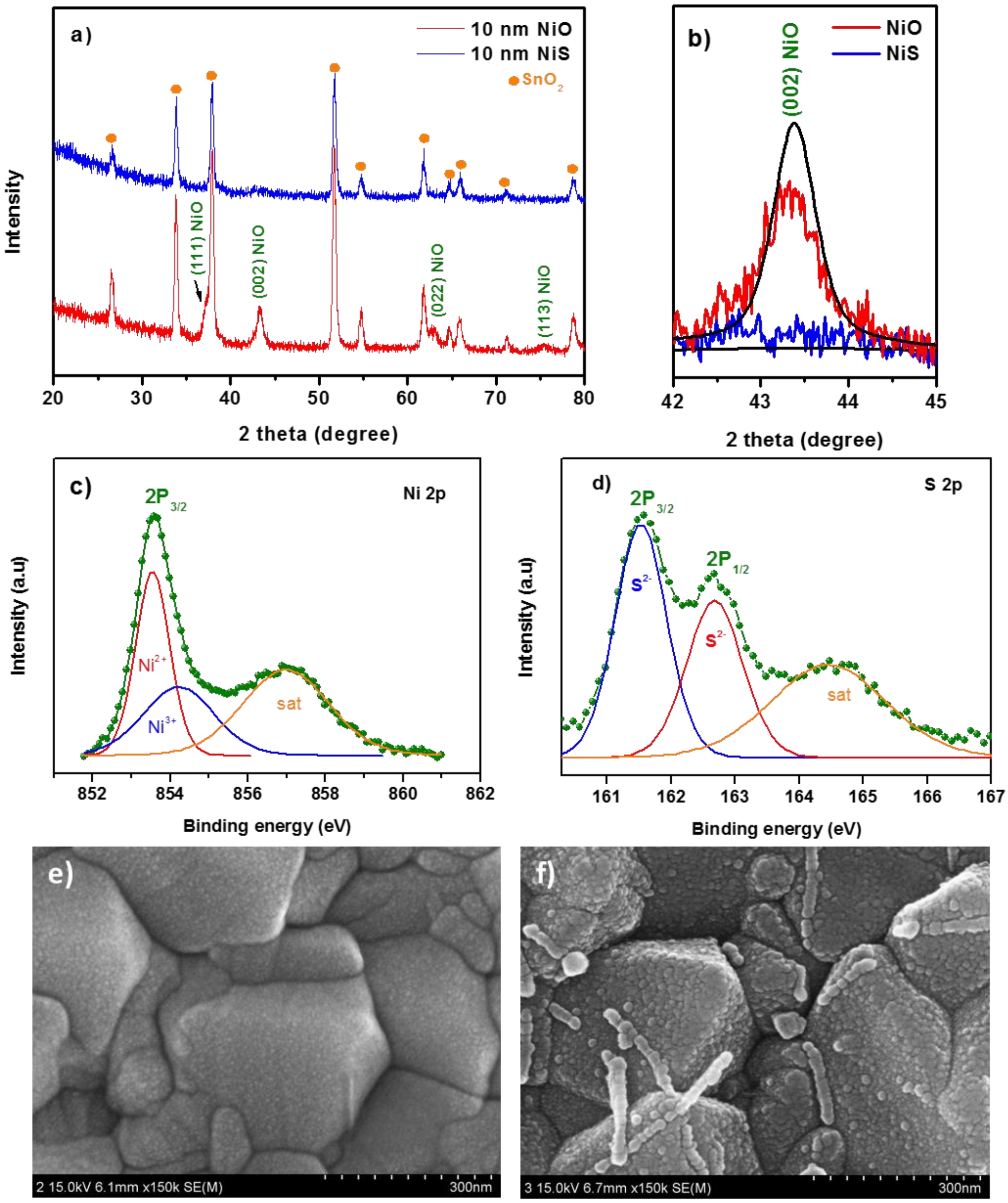

Fig. S1 (**a**) XRD spectra of as-deposited 10 nm NiO thin films and ion-exchange-processed NiS thin films. (**b**) Enlarged view of the peak corresponding to the (002) plane of NiO before and after ion exchange. High-resolution XPS (**c**) Ni 2p and (**d**) S 2p spectra of the NiS film. FE-SEM images of (**e**) as-deposited 10 nm NiO thin film and (**f**) NiS film

As shown in Fig. S1(a), the 10 nm NiO thin films grown by ALD on FTO show a strong NiO fingerprint at 44.27°, which corresponds to the (002) diffraction plane. Further, three additional peaks at 37.27°, 62.96°, and 75.54°, which correspond to the (111), (022), and (113) planes of NiO, respectively, are observed. Unfortunately, the sulfurized NiO film does not show any characteristic peaks of NiS, likely because of the amorphous nature of the formed NiS thin film. However, the NiO peaks completely disappeared after the anion exchange process, which confirms that the O^{2-} ions in the NiO lattice were liberated by S^{2-} ions. Figure S1(b) shows the disappearance of the peak corresponding to the (002) plane of NiO after the anion exchange process. The deconvoluted peaks of the Ni $2p_{3/2}$ orbital are shown in Fig. S1(c); the spectrum was fitted with two peaks representing the Ni^{2+} and Ni^{3+} states. The minor amount of Ni^{3+} may be attributed to the formation of a Ni_3S_4 phase during the ion exchange process. The S2p spectra were deconvoluted into the $2p_{3/2}$ and $2p_{1/2}$ spin orbitals at approximately 161.6 and 162.4 eV, respectively. In the survey spectrum, the S^{2-} state at 161.52 and 162.7 eV is dominant for the $2p_{3/2}$ and $2p_{1/2}$ orbitals, respectively [Fig. S1(d)]. The molar ratio of S/Ni was 1.02. The morphological changes in the NiO films after the anion exchange process were analyzed using FE-SEM. Figure S1(e) shows the 10 nm NiO layer coated on the FTO substrate; a highly conformal film was formed over the SnO_2 crystals. In this case, the NiO film thickness is too small, and therefore, we can assume that ion exchange occurs via dissolution/recrystallization. Briefly, NiO is dissolved in the S source owing to the difference in solubility product constant. The complete dissolution of NiO produced Ni^{2+} ions in the solution, leaving behind surface-anchored residual Ni atoms. Next, NiS was formed by the reaction between dissolved Ni^{2+} and S^{2-} ions in the solution, which also occurred simultaneously on the surface by homogenous and heterogeneous nucleation, respectively. With increasing time, nuclei on the surface began to grow into distinct islands [Fig. S1(f)]. The dissolution/recrystallization process resulted in a significant loss of Ni ions into the solution owing to the homogeneous nucleation of NiS in the solution phase. EDS analysis revealed that the atomic percentages of Ni and S are 55.47% and 44.53%, respectively, which are very close to the stoichiometry of NiS; further, these results agreed well with the XPS results. These results indicate that the converted NiO films contain NiS as the major constituent, along with minute quantities of S-rich nickel sulfides as impurities.

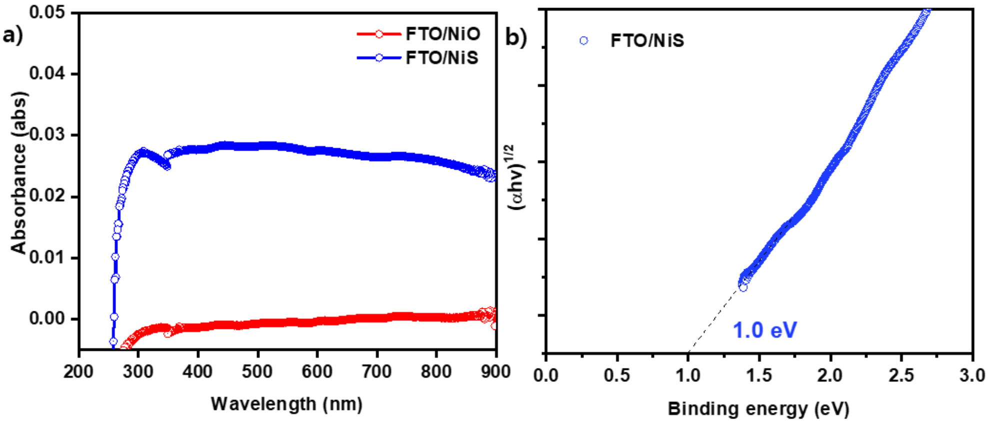

Fig. S2 (**a**) UV–vis spectra of NiO (10 nm)-coated FTO before and after anion exchange reaction, (**b**) corresponding Tauc plot

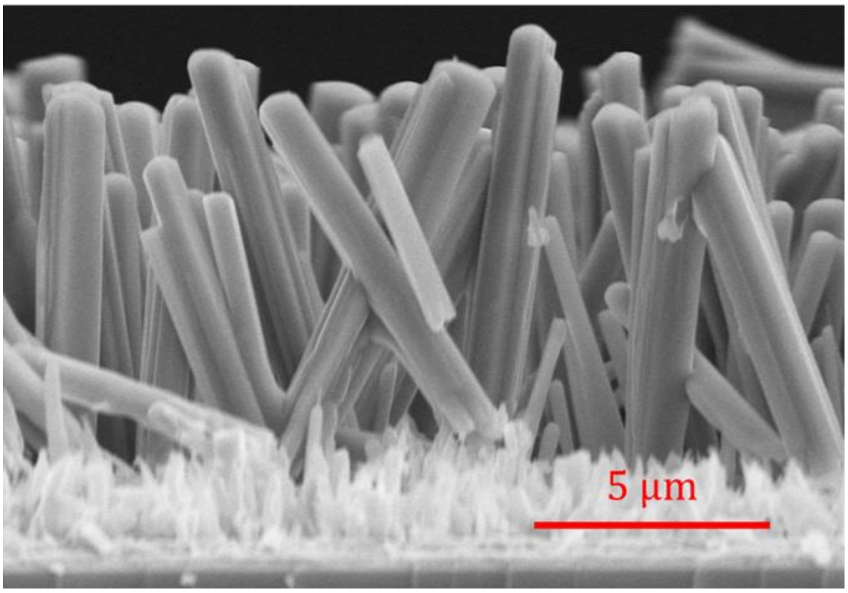

Fig. S3 Cross-sectional HR-SEM image of Bi_2S_3 photoanode

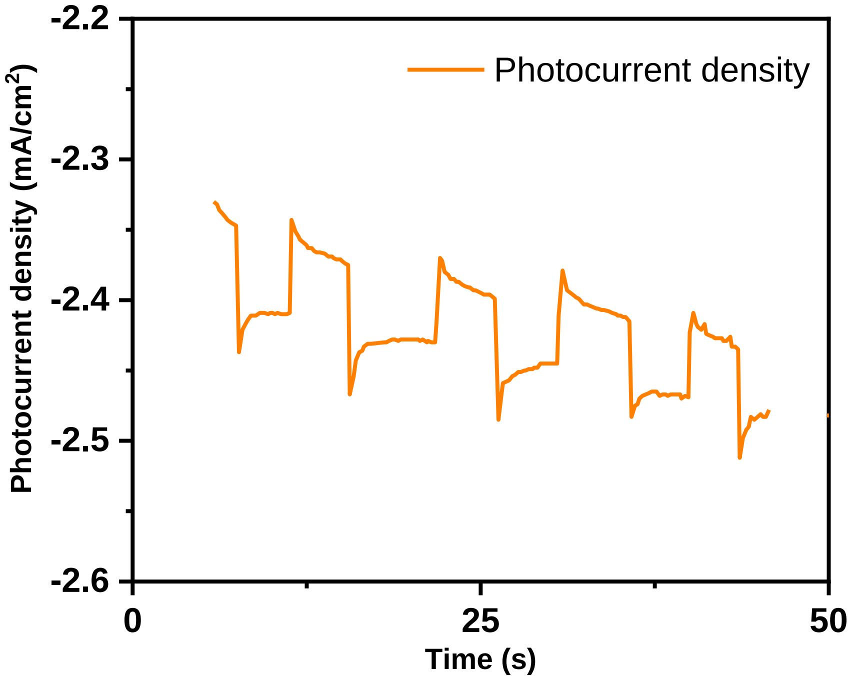

Fig. S4 *J–T* curve of NiS photocathode in 0.25 M Na_2S electrolyte at 0.0 V under illumination

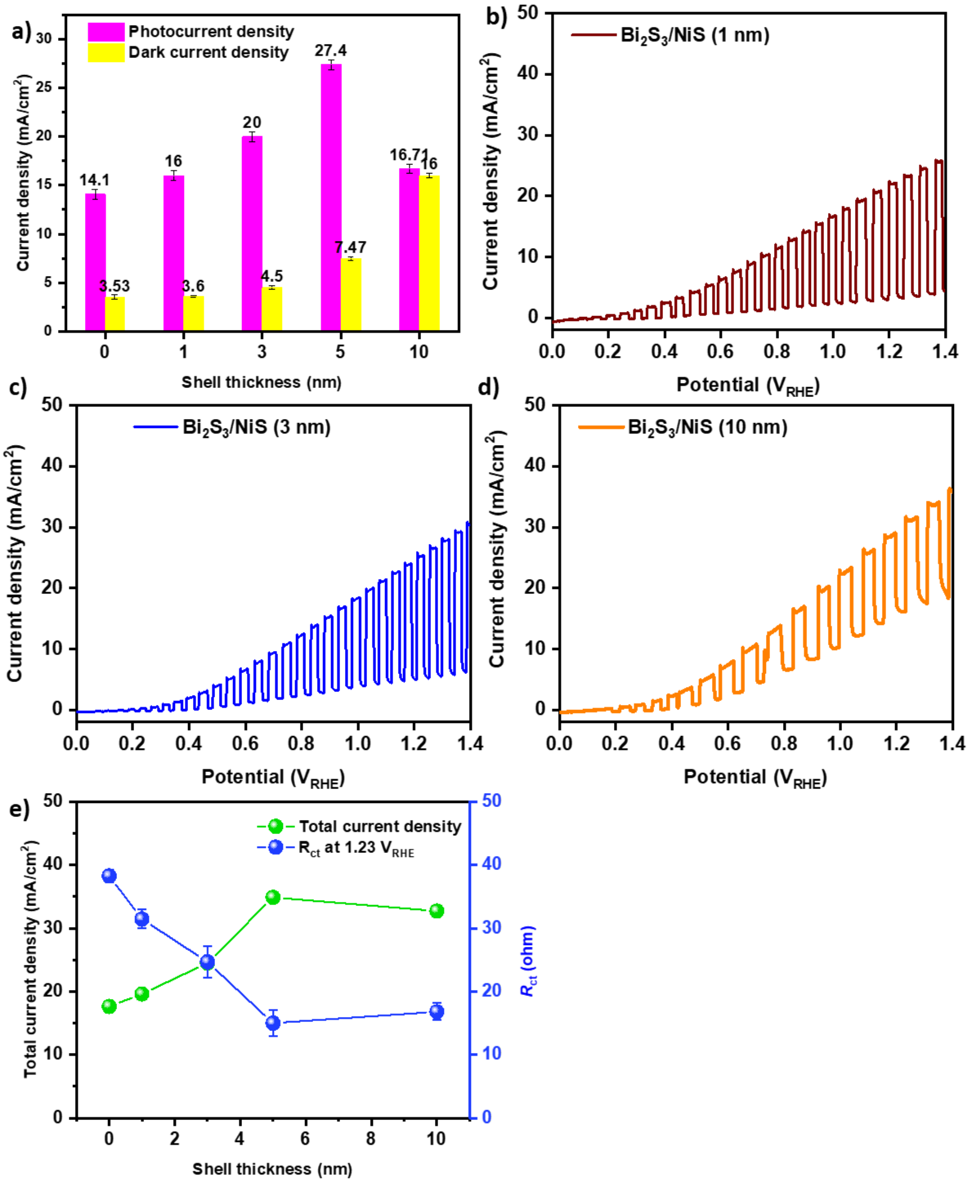

Fig. S5 (**a**) Comparison of photocurrent and dark current measured with shell layers of different thickness obtained from *J*–*V* curves shown in (**b**–**d**), and (**e**) Comparison of total current density and charge transfer resistance (R_{ct}) at 1.23 V_{RHE}

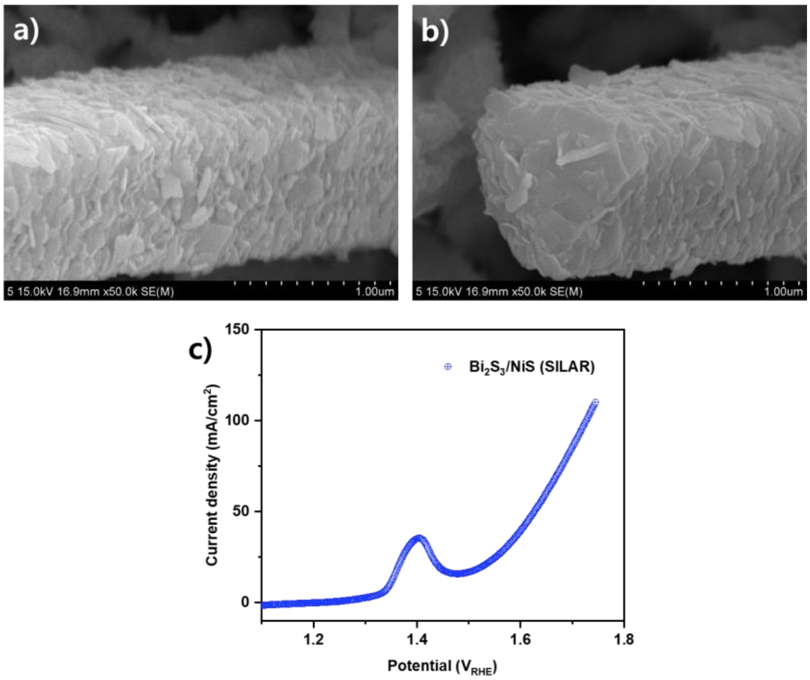

Fig. S6 (**a, b**) HR-SEM images of Bi_2S_3/NiS (SILAR), (**c**) *J–V* curve of Bi_2S_3/NiS (SILAR) in 0.25 M Na_2S electrolyte under chopped illumination

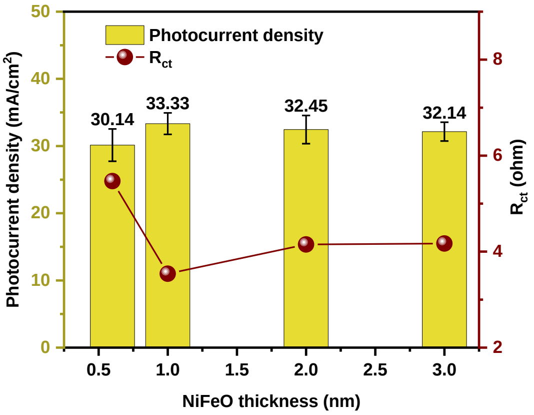

Fig. S7 Comparison of photocurrent density and charge transfer resistance (R_{ct}) values (at 0.7 V_{RHE}) measured with different NiFeO thicknesses in 0.25 M Na_2S electrolyte under illumination

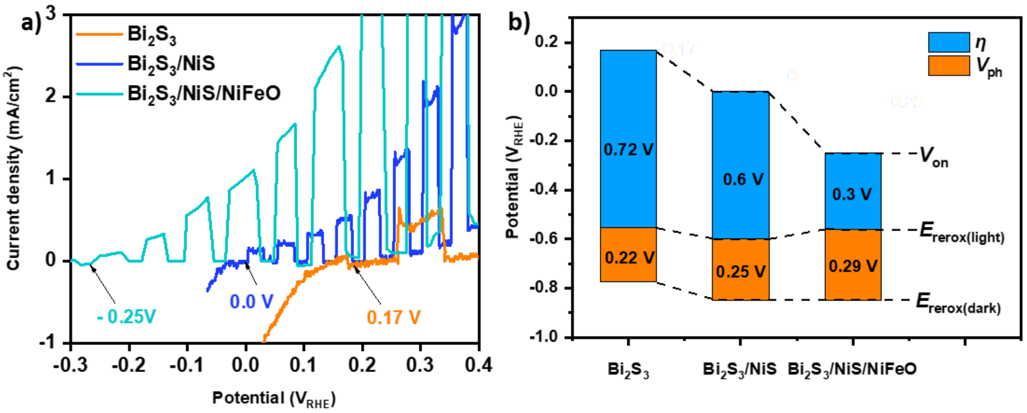

Fig. S8 (**a**) Photocurrent onset (V_{on}) of photoanodes under chopped illumination in 0.25 M Na_2S electrolyte, and (**b**) calculated photovoltage (V_{ph}) and kinetic overpotential (η) values for each photoanode

Calculation: The detailed calculations for each parameter are shown below,

$$E_{redox(dark)} - V_{on} = V_{ph} - \eta \quad \text{(S1)}$$

$$V_{ph} = | E_{redox(dark)} - E_{redox(light)} | \quad \text{(S2)}$$

$$\eta = |E_{redox(light)} - V_{on}| \quad \text{(S3)}$$

The values of $E_{\text{redox(dark)}}$ and $E_{\text{redox(light)}}$ are obtained from Fig. 3(b), V_{on} is obtained from Fig. S8(a), and η is the potential difference between $E_{\text{redox(light)}}$ and V_{on}. All values calculated for each photoanode are listed in Table S3.

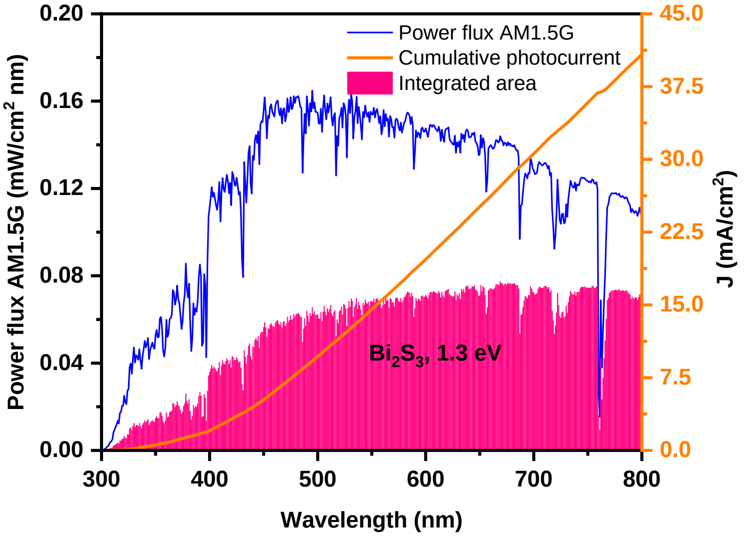

Fig. S9 Light-harvesting efficiency of Bi_2S_3/NiS/NiFeO corresponding to AM 1.5 G spectrum. Absorption photocurrent (J_{abs}) is 41.25 mA/cm^2

Calculation: The experimentally obtained photocurrent densities are much lower than the theoretical maximum value (J_{max} = 41.25 mA cm^{-1}) calculated for a band gap of 1.3 eV [S1]. We calculated the efficiencies of each step (photon absorption, η_{abs}; charge separation, η_{bulk}; charge injection, η_{surface}) to determine the major factors limiting the PEC performance [S2].

$$J_{\text{PEC}} = J_{\text{max}} \times \eta_{\text{abs}} \times \eta_{\text{bulk}} \times \eta_{\text{surface}} \quad \text{(S4)}$$

$$\eta_{\text{abs}} = J_{\text{abs}}/J_{\text{max}} \quad \text{(S5)}$$

$$\eta_{\text{sep}} = J_{\text{Na2S}}/J_{\text{abs}} \quad \text{(S6)}$$

The absorption efficiency, η_{abs}, is a quantitative indicator of the fraction of photons absorbed from the total solar flux; it can be calculated by determining the absorption photocurrent density, J_{abs}, and integrating the solar photon flux within the wavelength range of the semiconductor [Fig. S11]. Thus, J_{abs} is the maximum photocurrent that can be obtained using the synthesized bismuth sulfide electrodes, assuming that η_{bulk} and η_{surface} are equal to unity. To estimate η_{bulk} and η_{surface} quantitatively, the photocurrent (J_{SO3}) was measured using 0.35 M Na_2SO_3 as a hole scavenger [S3].

$$\eta_{\text{inj}} = \frac{J_{\text{Na2S}}}{J_{\text{SO3}}} \quad \text{(S7)}$$

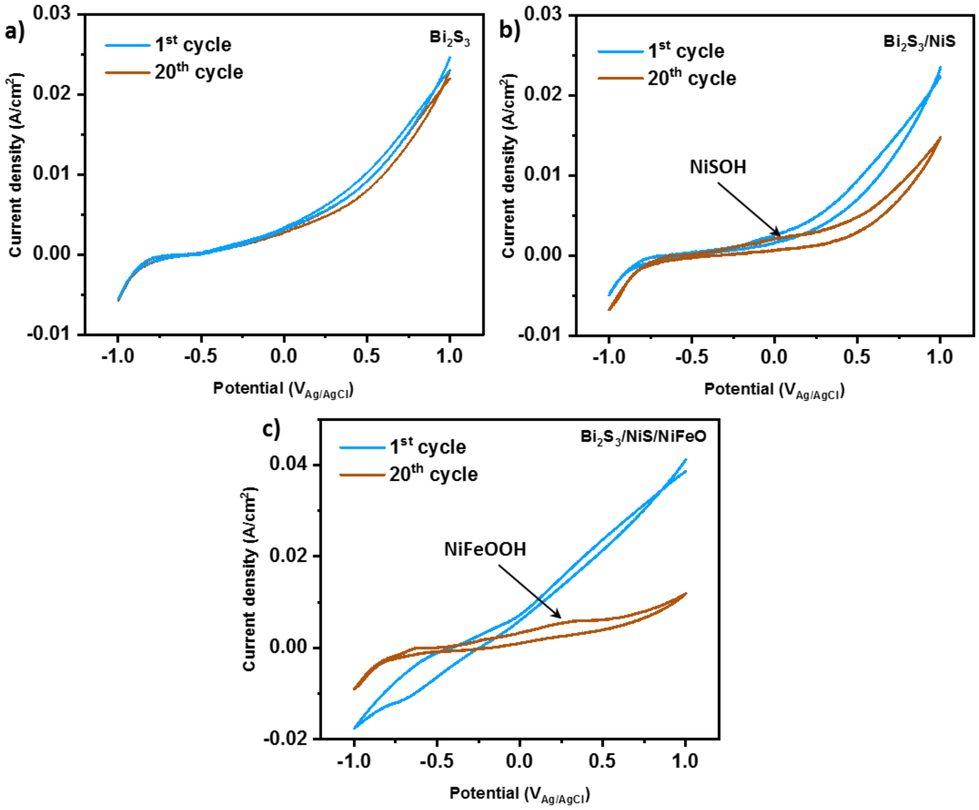

Fig. S10 Cyclic voltammetry curves of Bi_2S_3/NiS and Bi_2S_3 photoanodes in 0.25 M Na_2S electrolyte under dark conditions

NiFe-based oxide/oxyhydroxide catalysts are electrolyte-permeable "volume catalysts" that enable direct contact between the electrolyte and catalyst shell, as well as the semiconductor core [S4]. In this study, ALD-deposited $NiFeO_x$ catalysts were converted to a layered hydroxide [$NiFe(OH)_2$] and then an electrolyte-permeable oxyhydroxide (NiFeOOH) during the oxidative redox reaction over consecutive anodic linear sweep voltammetry cycles [$M(OH)_2 + OH^- \rightarrow MOOH + H_2O + e^-$] [S5]. The redox peak at 0.35 $V_{Ag/AgCl}$ confirms the formation of $Ni^{2+/3+}$ and $Fe^{3+/4+}$ redox couples.

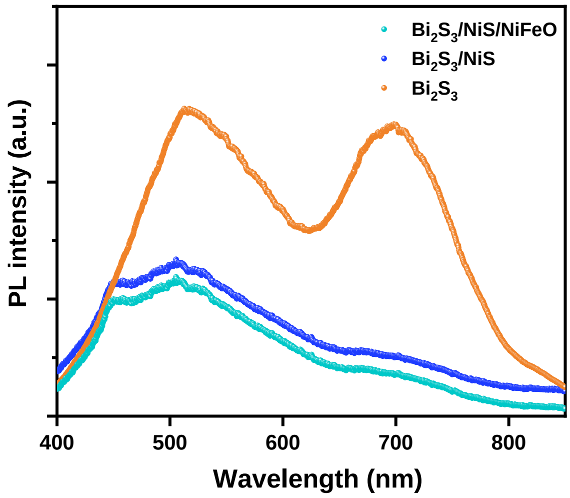

Fig. S11 Photoluminescence of Bi_2S_3, Bi_2S_3/NiS, and Bi_2S_3/NiS/NiFeO photoanodes

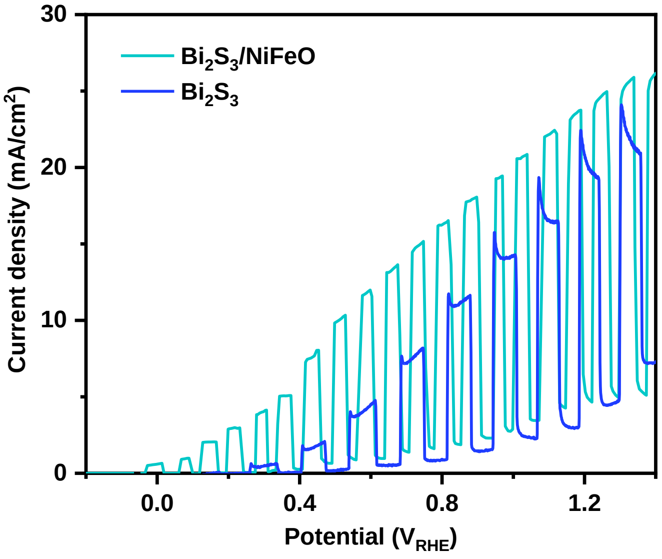

Fig. S12 *J*–*V* curves of Bi_2S_3 and Bi_2S_3/NiFeO photoanodes in 0.25 M Na_2S electrolyte

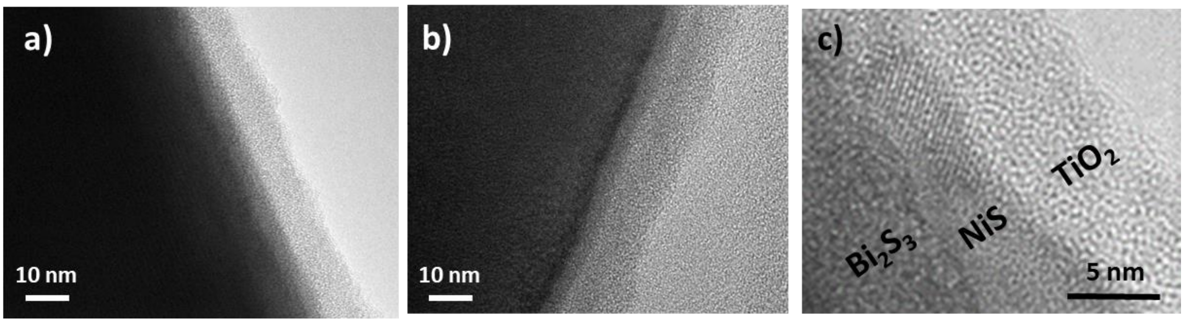

Fig. S13 (**a-c**) HRTEM image of Bi_2S_3/NiS/NiFeO/TiO_2 photoanode

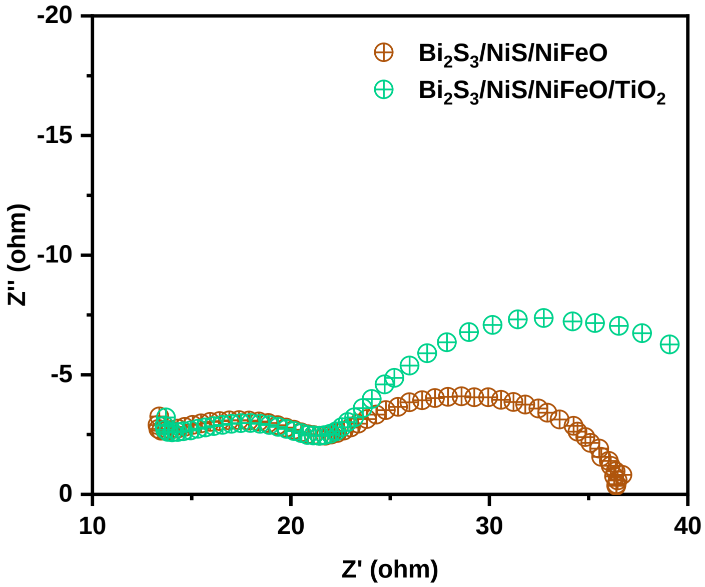

Fig. S14 Comparison of Nyquist plots of Bi_2S_3/NiS/NiFeO and Bi_2S_3/NiS/NiFeO/TiO_2 photoanode at 0.7 V_{RHE} under illumination in 0.25 M Na_2S electrolyte

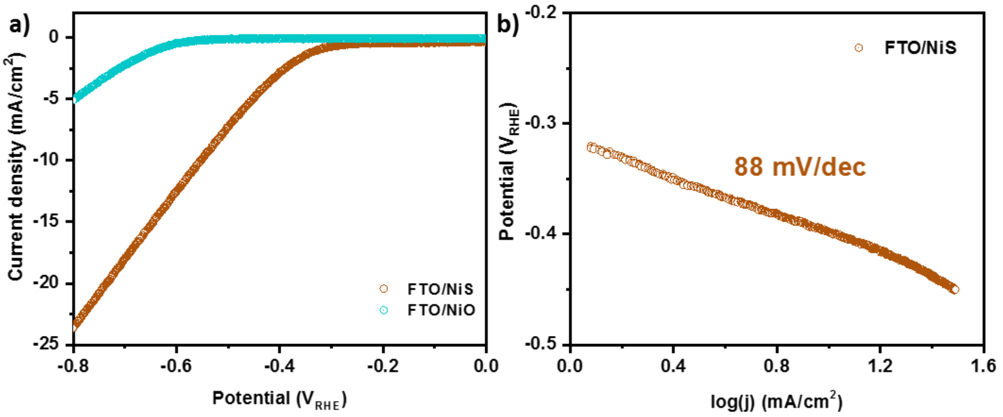

Fig. S15 (**a**) *iR*-uncorrected linear sweep voltammetry of FTO/NiO and FTO/NiS electrodes for HER polarization in 0.25 M Na_2S (pH ~ 12.5) and (**b**) corresponding Tafel plot

The NiS films were tested in the same manner as described earlier for the oxygen evolution reaction, but in a different potential range from 0 to −0.8 V_{RHE}. As shown in Fig. S13(a), the NiS film with a Pt counter electrode shows an overpotential of 396 mV at a current density of 10 mA cm^{-2}. The corresponding Tafel slope (88 mV dec^{-1}) reveals that the hydrogen evolution reaction (HER) proceeds along the Volmer–Heyrovsky pathway, where the Heyrovsky step is the rate-determining step.

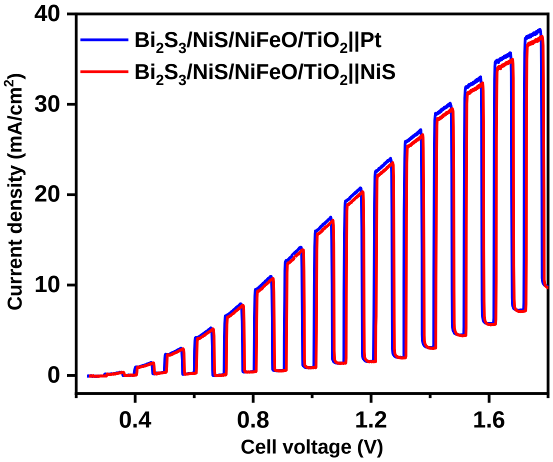

Fig. S16 *J*–*V* curve of Bi_2S_3/NiS/NiFeO/TiO_2 photoanode with Pt and NiS electrocathode in integrated PEC-EC water splitting cell

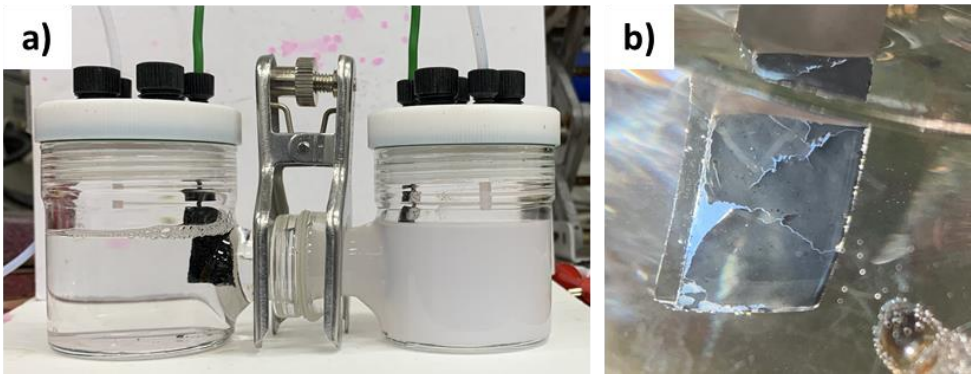

Fig. S17 (**a**) Photographs of H cell used for long term stability and gas chromatography tests, (**b**) photographs of Bi_2S_3/NiS/NiFeO photoanode after chronopotentiometry test shown in **Fig. 6(g)**

Table S1 Comparison of recent reports on hematite photoanodes majorly with NiFe incorporated OER catalyst

No.	Photoanode	Electrolyte	Photocurrent density at 1.23 V_{RHE} (mA cm^{-2})	Refs.
1	Bi_2S_3/NiS/NiFeO	0.25 M Na_2S (pH=12.5)	33.35	Our work
		0.5 M Na_2SO_4 (pH=7.0)	10.44	
2	2D/1D In_2S_3@Bi_2S_3	0.2 M Na_2SO_4 (pH=6.8)	2.0	[S6]
3	$BiVO_4$/Bi_2S_3	0.25 M Na_2S + 0.35 M Na_2SO_3 (pH=11.5)	7.81 at 0.97 V_{RHE}	[S7]
4	WO_3/Bi_2S_3	0.1 M Na_2S + 0.1 M Na_2SO_3 (pH=12)	6.56 at 0.9 V_{RHE}	[S8]
5	Mo-WO_3/Fe-WO_3/Bi_2S_3	0.2 M Na_2SO_4 (pH=6.8)	2.55	[S9]
6	$BiVO_4$/Bi_2S_3	Na_2S + Na_2SO_3	3.30	[S10]
7	PANI/Bi_2S_3	0.5 M Na_2SO_4 (pH=7.0)	4.97	[S11]
8	porous Ni-O/Ni/pn^+-Si	1 M NaOH (pH=13.5)	39.7	[S12]
9	Ni/TiO2/p^+n-Si		33.6	[S13]
10	$CdIn_2S_4$	0.25 M Na_2S + 0.35 M Na_2SO_3 (pH=12.5)	5.73	[S14]
11	Ti-WS_2	0.05 M Na_2S	10.44	[S15]
12	$CdIn_2S_4$/In_2S_3	0.5 M Na_2SO_4 (pH=7.0)	2.98	[S16]
13	$ZnIn_2S_4$	0.5 M Na_2HPO_4/NaH_2PO_4 (pH = 6.5)	3.52	[S17]
14	Bi_2O_3/Bi_2S_3	0.1 M Na_2S + 0.1 M Na_2SO_3 (pH=12.5)	9.7 at 0.80 V_{RHE}	[S18]
15	rGO/Bi_2S_3		6.06	[S19]
16	MoS_2/CdS/TiO_2	0.25 M Na_2S + 0.35 M Na_2SO_3 (pH=12.5)	3.25 at 0.90 V_{RHE}	[S20]
17	$Cd_xZn_{1-x}S$/ZnO		6.6 at 0 $V_{Ag/AgCl}$	[S21]
18	$CdIn_2S_4$/TiO_2		3.88 at 0 $V_{Ag/AgCl}$	[S22]
19	Co-Pi/$ZnInS_4$/Pt		0.91	[S23]
20	PbS/CdS/ZnO	0.5 M Na_2S + 0.5 M Na_2SO_3 (pH=12.5)	14.2 at 0 $V_{Ag/AgCl}$	[S24]

Table S2 Atomic percentage of each elements from XPS measurements of Bi_2S_3/NiS/NiFeO/TiO_2 photoanode after 4 h of photo-electrolysis

	At %
S 2p	8.96
O 1s	44.31
Mg 2p	0.24
Ca 2p	1.04
Cl 2p	0.46

Table S3 Calculated photovoltage (V_{ph}) and kinetic overpotential (η) for all photoanodes

Photoanodes	$E_{redox(dark)}$	$E_{redox(light)}$	V_{on}	V_{ph}	η
Bi_2S_3	–0.77 V_{RHE}	–0.55 V_{RHE}	0.17 V_{RHE}	0.22 V	0.72 V
Bi_2S_3/NiS	–0.85 V_{RHE}	–0.60 V_{RHE}	0.00 V_{RHE}	0.25 V	0.60 V
Bi_2S_3/NiS/NiFeO	–0.85 V_{RHE}	–0.56 V_{RHE}	–0.25 V_{RHE}	0.29 V	0.30 V

Supplementary References

[S1] Y.M. Ma, S.R. Pendlebury, A. Reynal, F.L. Formal, J.R. Durrant, Dynamics of photogenerated holes in undoped $BiVO_4$ photoanodes for solar water oxidation. Chem. Sci. **5**(8), 2964-2973 (2014). https://doi.org/10.1039/c4sc00469h

[S2] H. Dotan, K. Sivula, M. Gratzel, A. Rothschild, S.C. Warren, Probing the photoelectrochemical properties of hematite (alpha-Fe_2O_3) electrodes using hydrogen peroxide as a hole scavenger. Energy Environ. Sci. **4**(3), 958-964 (2011). https://doi.org/10.1039/c0ee00570c

[S3] S. Selvaraj, H. Moon, D.H. Kim, Combined effect of nano-structured $NiCo_2S_4$ coated hematite photoanodes for efficient photoelectrochemical water oxidation. Catal. Today **347**, 63-69 (2018). https://doi.org/10.1016/j.cattod.2018.05.045

[S4] M.R. Nellist, F.A.L. Laskowski, F. Lin, T.J. Mills, S.W. Boettcher, Semiconductor–electrocatalyst interfaces: theory, experiment, and applications in photoelectrochemical water splitting. Acc. Chem. Res. **49**(4), 733-740 (2016). https://doi.org/10.1021/acs.accounts.6b00001

[S5] M.Y. Li, T. Liu, Y. Yang, W.T. Qiu, C.L. Liang et al., Zipping up $NiFe(OH)_x$-encapsulated hematite to achieve an ultralow turn-on potential for water oxidation. ACS Energy Lett. **4**(8), 1983-1990 (2019). https://doi.org/10.1021/acsenergylett.9b01430

[S6] Y. Xiong, L. Yang, D.K. Nandakumar, Y. Yang, H. Dong et al., Highly efficient photoelectrochemical water oxidation enabled by enhanced interfacial interaction in 2D/1D In_2S_3@Bi_2S_3 heterostructures. J. Mater. Chem. A **8**(11), 5612-5621 (2020). https://doi.org/10.1039/D0TA00149J

[S7] C. Liu, J. Li, Y. Li, W. Li, Y. Yang et al., Epitaxial growth of Bi_2S_3 nanowires on $BiVO_4$ nanostructures for enhancing photoelectrochemical performance. RSC Adv. **5**(88), 71692-71698 (2015). https://doi.org/10.1039/C5RA13171E

[S8] Y. Wang, W. Tian, L. Chen, F. Cao, J. Guo et al., Three-dimensional WO_3 nanoplate/Bi_2S_3 nanorod heterojunction as a highly efficient photoanode for improved photoelectrochemical water splitting. ACS Appl. Mater. Interfaces **9**(46), 40235-40243 (2017). https://doi.org/10.1021/acsami.7b11510

[S9] Y. Li, Z. Liu, J. Li, M. Ruan, Z. Guo, An effective strategy of constructing a multi-junction structure by integrating a heterojunction and a homojunction to promote the charge separation and transfer efficiency of WO_3. J. Mater. Chem. A **8**(13), 6256-6267 (2020). https://doi.org/10.1039/D0TA00452A

[S10] M.A. Mahadik, H.S. Chung, S.Y. Lee, M. Cho, J.S. Jang, In-situ noble fabrication of Bi_2S_3/$BiVO_4$ hybrid nanostructure through a photoelectrochemical transformation process for solar hydrogen production. ACS Sustain. Chem. Eng. **6**(9), 12489-12501 (2018). https://doi.org/10.1021/acssuschemeng.8b03140

[S11] S. Sharma, D. Kumar, N. Khare, Three-dimensional hierarchical PANI/Bi_2S_3 nanoflowers heterojunction for enhanced photoelectrochemical water splitting. J. Alloys Compd. **865**, 158779 (2021). https://doi.org/10.1016/j.jallcom.2021.158779

[S12] G. Huang, R. Fan, X. Zhou, Z. Xu, W. Zhou et al., A porous Ni-O/Ni/Si photoanode for stable and efficient photoelectrochemical water splitting. Chem. Commun. **55**(3), 377-380 (2019). https://doi.org/10.1039/C8CC08146H

[S13] S. Hu, M.R. Shaner, J.A. Beardslee, M. Lichterman, B.S. Brunschwig et al., Amorphous TiO_2 coatings stabilize Si, GaAs, and gap photoanodes for efficient water oxidation.

Science **344**(6187), 1005-1009 (2014). https://doi.org/10.1126/science.1251428

[S14] H. Wang, Y. Xia, H. Li, X. Wang, Y. Yu et al., Highly active deficient ternary sulfide photoanode for photoelectrochemical water splitting. Nat. Commun. **11**, 3078 (2020). https://doi.org/10.1038/s41467-020-16800-w

[S15] A. Ahmadi, M.Z. Shoushtari, Enhancing the photoelectrochemical water splitting performance of WS_2 nanosheets by doping titanium and molybdenum via a low temperature CVD method. J. Electroanal. Chem. **849**, 113361 (2019). https://doi.org/10.1016/j.jelechem.2019.113361

[S16] L. Meng, M. Wang, H. Sun, W. Tian, C. Xiao et al., Designing a transparent $CdIn_2S_4/In_2S_3$ bulk-heterojunction photoanode integrated with a perovskite solar cell for unbiased water splitting. Adv. Mater. **32**(30), 2002893 (2020). https://doi.org/10.1002/adma.202002893

[S17] W. Xu, W. Gao, L. Meng, W. Tian, L. Li, Incorporation of sulfate anions and sulfur vacancies in $ZnIn_2S_4$ photoanode for enhanced photoelectrochemical water splitting. Adv. Energy Mater. **11**(26), 2101181 (2021). https://doi.org/10.1002/aenm.202101181

[S18] J.H. Kim, T. Lim, J.Y. Park, A. Ma, H. Jung et al., Understanding and improving photoelectrochemical performance of Bi_2O_3/Bi_2S_3 composite. New J. Chem. **43**(30), 11893-11902 (2019). https://doi.org/10.1039/C9NJ02913C

[S19] P. Subramanyam, B. Meena, G.N. Sinha, D. Suryakala, C. Subrahmanyam, Facile synthesis and photoelectrochemical performance of a Bi_2S_3@rGO nanocomposite photoanode for efficient water splitting. Energy Fuels **35**(7), 6315-6321 (2021). https://doi.org/10.1021/acs.energyfuels.1c00084

[S20] S.S.M. Bhat, S.A. Pawar, D. Potphode, C.K. Moon, J.M. Suh et al., Substantially enhanced photoelectrochemical performance of TiO_2 nanorods/CdS nanocrystals heterojunction photoanode decorated with MoS_2 nanosheets. Appl. Catal. B Environ. **259**, 118102 (2019). https://doi.org/10.1016/j.apcatb.2019.118102

[S21] R.P. Patil, M.A. Mahadik, W.S. Chae, S.H. Choi, J.S. Jang, Self-templated fabrication of 2-D dual nanoarchitecture $Zn_{1-x}Cd_xS$ porous nanosheet and ZnO nanorod for photoelectrochemical hydrogen production. Appl. Surf. Sci. **539**, 148267 (2021). https://doi.org/10.1016/j.apsusc.2020.148267

[S22] L.K. Dhandole, M.A. Mahadik, H.S. Chung, W.S. Chae, M. Cho et al., $CdIn_2S_4$ chalcogenide/TiO_2 nanorod heterostructured photoanode: an advanced material for photoelectrochemical applications. Appl. Surf. Sci. **490**, 18-29 (2019). https://doi.org/10.1016/j.apsusc.2019.05.222

[S23] M. Zhou, Z. Liu, Q. Song, X. Li, B. Chen et al., Hybrid 0D/2D edamame shaped $ZnIn_2S_4$ photoanode modified by Co-Pi and Pt for charge management towards efficient photoelectrochemical water splitting. Appl. Catal. B Environ. **244**, 188-196 (2019). https://doi.org/10.1016/j.apcatb.2018.11.031

[S24] R. Wang, S. Chen, Y.H. Ng, Q. Gao, S. Yang et al., ZnO/CdS/PbS nanotube arrays with multi-heterojunctions for efficient visible-light-driven photoelectrochemical hydrogen evolution. Chem. Eng. J. **362**, 658-666 (2019). https://doi.org/10.1016/j.cej.2019.01.073

[S25] Y.M. Ma, S.R. Pendlebury, A. Reynal, F.L. Formal, J.R. Durrant, Dynamics of photogenerated holes in undoped $BiVO_4$ photoanodes for solar water oxidation. Chem. Sci. **5**(8), 2964-2973 (2014). https://doi.org/10.1039/c4sc00469h

[S26] H. Dotan, K. Sivula, M. Gratzel, A. Rothschild, S.C. Warren, Probing the

photoelectrochemical properties of hematite (alpha-Fe_2O_3) electrodes using hydrogen peroxide as a hole scavenger. Energy Environ. Sci. **4**(3), 958-964 (2011). https://doi.org/10.1039/c0ee00570c

[S27] S. Selvaraj, H. Moon, D.H. Kim, Combined effect of nano-structured $NiCo_2S_4$ coated hematite photoanodes for efficient photoelectrochemical water oxidation. Catal. Today **347**, 63-69 (2018). https://doi.org/10.1016/j.cattod.2018.05.045

[S28] M.R. Nellist, F.A.L. Laskowski, F. Lin, T.J. Mills, S.W. Boettcher, Semiconductor–electrocatalyst interfaces: theory, experiment, and applications in photoelectrochemical water splitting. Acc. Chem. Res. **49**(4), 733-740 (2016). https://doi.org/10.1021/acs.accounts.6b00001

[S29] M.Y. Li, T. Liu, Y. Yang, W.T. Qiu, C.L. Liang et al., Zipping up $NiFe(OH)_x$-encapsulated hematite to achieve an ultralow turn-on potential for water oxidation. ACS Energy Lett. **4**(8), 1983-1990 (2019). https://doi.org/10.1021/acsenergylett.9b01430

[S30] Y. Xiong, L. Yang, D.K. Nandakumar, Y. Yang, H. Dong et al., Highly efficient photoelectrochemical water oxidation enabled by enhanced interfacial interaction in 2D/1D In_2S_3@Bi_2S_3 heterostructures. J. Mater. Chem. A **8**(11), 5612-5621 (2020). https://doi.org/10.1039/D0TA00149J

[S31] C. Liu, J. Li, Y. Li, W. Li, Y. Yang et al., Epitaxial growth of Bi_2S_3 nanowires on $BiVO_4$ nanostructures for enhancing photoelectrochemical performance. RSC Adv. **5**(88), 71692-71698 (2015). https://doi.org/10.1039/C5RA13171E

[S32] Y. Wang, W. Tian, L. Chen, F. Cao, J. Guo et al., Three-dimensional WO_3 nanoplate/Bi_2S_3 nanorod heterojunction as a highly efficient photoanode for improved photoelectrochemical water splitting. ACS Appl. Mater. Interfaces **9**(46), 40235-40243 (2017). https://doi.org/10.1021/acsami.7b11510

[S33] Y. Li, Z. Liu, J. Li, M. Ruan, Z. Guo, An effective strategy of constructing a multi-junction structure by integrating a heterojunction and a homojunction to promote the charge separation and transfer efficiency of WO_3. J. Mater. Chem. A **8**(13), 6256-6267 (2020). https://doi.org/10.1039/D0TA00452A

[S34] M.A. Mahadik, H.S. Chung, S.Y. Lee, M. Cho, J.S. Jang, In-situ noble fabrication of Bi_2S_3/$BiVO_4$ hybrid nanostructure through a photoelectrochemical transformation process for solar hydrogen production. ACS Sustain. Chem. Eng. **6**(9), 12489-12501 (2018). https://doi.org/10.1021/acssuschemeng.8b03140

[S35] S. Sharma, D. Kumar, N. Khare, Three-dimensional hierarchical PANI/Bi_2S_3 nanoflowers heterojunction for enhanced photoelectrochemical water splitting. J. Alloys Compd. **865**, 158779 (2021). https://doi.org/10.1016/j.jallcom.2021.158779

[S36] G. Huang, R. Fan, X. Zhou, Z. Xu, W. Zhou et al., A porous Ni-O/Ni/Si photoanode for stable and efficient photoelectrochemical water splitting. Chem. Commun. **55**(3), 377-380 (2019). https://doi.org/10.1039/C8CC08146H

[S37] S. Hu, M.R. Shaner, J.A. Beardslee, M. Lichterman, B.S. Brunschwig et al., Amorphous TiO_2 coatings stabilize Si, GaAs, and gap photoanodes for efficient water oxidation. Science **344**(6187), 1005-1009 (2014). https://doi.org/10.1126/science.1251428

[S38] H. Wang, Y. Xia, H. Li, X. Wang, Y. Yu et al., Highly active deficient ternary sulfide photoanode for photoelectrochemical water splitting. Nat. Commun. **11**(1), 3078 (2020). https://doi.org/10.1038/s41467-020-16800-w

[S39] A. Ahmadi, M.Z. Shoushtari, Enhancing the photoelectrochemical water splitting

performance of WS_2 nanosheets by doping titanium and molybdenum via a low temperature CVD method. J. Electroanal. Chem. **849**, 113361 (2019). https://doi.org/10.1016/j.jelechem.2019.113361

[S40] L. Meng, M. Wang, H. Sun, W. Tian, C. Xiao et al., Designing a transparent $CdIn_2S_4/In_2S_3$ bulk-heterojunction photoanode integrated with a perovskite solar cell for unbiased water splitting. Adv. Mater. **32**(30), 2002893 (2020). https://doi.org/10.1002/adma.202002893

[S41] W. Xu, W. Gao, L. Meng, W. Tian, L. Li, Incorporation of sulfate anions and sulfur vacancies in $ZnIn_2S_4$ photoanode for enhanced photoelectrochemical water splitting. Adv. Energy Mater. **11**(26), 2101181 (2021). https://doi.org/10.1002/aenm.202101181

[S42] J.H. Kim, T. Lim, J.Y. Park, A. Ma, H. Jung et al., Understanding and improving photoelectrochemical performance of Bi_2O_3/Bi_2S_3 composite. New J. Chem. **43**(30), 11893-11902 (2019). https://doi.org/10.1039/C9NJ02913C

[S43] P. Subramanyam, B. Meena, G.N. Sinha, D. Suryakala, C. Subrahmanyam, Facile synthesis and photoelectrochemical performance of a Bi_2S_3@rGO nanocomposite photoanode for efficient water splitting. Energy Fuels **35**(7), 6315-6321 (2021). https://doi.org/10.1021/acs.energyfuels.1c00084

[S44] S.S.M. Bhat, S.A. Pawar, D. Potphode, C.K. Moon, J.M. Suh et al., Substantially enhanced photoelectrochemical performance of TiO_2 nanorods/CdS nanocrystals heterojunction photoanode decorated with MoS_2 nanosheets. Appl. Catal. B Environ. **259**, 118102 (2019). https://doi.org/10.1016/j.apcatb.2019.118102

[S45] R.P. Patil, M.A. Mahadik, W.S. Chae, S.H. Choi, J.S. Jang, Self-templated fabrication of 2-D dual nanoarchitecture $Zn_{1-x}Cd_xS$ porous nanosheet and ZnO nanorod for photoelectrochemical hydrogen production. App. Surf. Sci. **539**, 148267 (2021). https://doi.org/10.1016/j.apsusc.2020.148267

[S46] L.K. Dhandole, M.A. Mahadik, H.S. Chung, W.S. Chae, M. Cho et al., $CdIn_2S_4$ chalcogenide/TiO_2 nanorod heterostructured photoanode: an advanced material for photoelectrochemical applications. Appl. Surf. Sci. **490**, 18-29 (2019). https://doi.org/10.1016/j.apsusc.2019.05.222

[S47] M. Zhou, Z. Liu, Q. Song, X. Li, B. Chen et al., Hybrid 0D/2D edamame shaped $ZnIn_2S_4$ photoanode modified by Co-Pi and Pt for charge management towards efficient photoelectrochemical water splitting. Appl. Catal. B Environ. **244**, 188-196 (2019). https://doi.org/10.1016/j.apcatb.2018.11.031

[S48] R. Wang, S. Chen, Y.H. Ng, Q. Gao, S. Yang et al., ZnO/CdS/PbS nanotube arrays with multi-heterojunctions for efficient visible-light-driven photoelectrochemical hydrogen evolution. Chem. Eng. J. **362**, 658-666 (2019). https://doi.org/10.1016/j.cej.2019.01.073
